# The Acute Effects of Non-concussive Head Impacts on Brain Microstructure, Chemistry and Function in Male Soccer Players: A Pilot Randomised Controlled Trial

**DOI:** 10.1186/s40798-025-00867-0

**Published:** 2025-06-18

**Authors:** Nathan Delang, Rebecca V. Robertson, Fernando A. Tinoco Mendoza, Luke A. Henderson, Caroline D. Rae, Stuart J. McDonald, Ben Desbrow, Christopher Irwin, Aimie L. Peek, Elizabeth A. Cairns, Paul J. Austin, Michael A. Green, Nicholas W. Jenneke, Jun Cao, William T. O’Brien, Shane Ball, Michael E. Buckland, Katherine Rae, Iain S. McGregor, Danielle McCartney

**Affiliations:** 1 School of Health Sciences and Social Work, Griffith University, Gold Coast, QLD Australia; 2 Queensland Academy of Sport, Nathan, QLD Australia; 3 School of Psychology, Faculty of Science, The University of Sydney, Sydney, NSW Australia; 4 Neuroscience Research Australia, Randwick, NSW Australia; 5 Brain and Mind Centre, The University of Sydney, Sydney, Australia; 6 School of Medical Sciences (Neuroscience), The University of Sydney, Sydney, Australia; 7 School of Psychology, The University of New South Wales, Kensington, NSW Australia; 8 Department of Neuroscience, Central Clinical School, Monash University, Melbourne, VIC Australia; 9 Department of Neurology, Alfred Hospital, Melbourne, VIC Australia; 10 Lambert Initiative for Cannabinoid Therapeutics, The University of Sydney, Sydney, NSW Australia; 11 Sydney Pharmacy School, The University of Sydney, Sydney, NSW Australia; 12 Faculty of Medicine and Health, School of Clinical Medicine, The University of New South Wales, Kensington, NSW Australia; 13 School of Biomedical Sciences, The University of New South Wales, Kensington, NSW Australia; 14 School of Health Sciences (Medicine & Health), The University of Sydney, Sydney, NSW Australia; 15 Department of Neuropathology, Royal Prince Alfred Hospital, Camperdown, NSW Australia; 16 The Sports Clinic, The University of Sydney, Sydney, NSW Australia; 17 Physical Preparation Department, Sydney Uni Sport and Fitness, Sydney, NSW Australia

**Keywords:** Subconcussion, Contact sport, Neural, Astroglial, Metabolites

## Abstract

**Background:**

Head impacts, particularly, *non-concussive* impacts, are common in sport. Yet, their effects on the brain remain poorly understood. Here, we investigated the acute effects of non-concussive impacts on brain microstructure, chemistry, and function using magnetic resonance imaging (MRI) and other techniques.

**Results:**

Fifteen healthy male soccer players participated in a randomised, controlled, crossover pilot trial. The intervention was a non-concussive soccer heading task (‘Heading’) and the control was an equivalent ‘Kicking’ task. Participants underwent MRI scans ~ 45 min post-task which took ~60 min to complete. Blood was also sampled, and cognitive function assessed, pre-, post-, 2.5 h post-, and 24 h post-task. Brain chemistry: Heading increased total *N*-acetylaspartate (*p* = 0.012; g = 0.66) and total creatine (*p* = 0.010; g = 0.77) levels in the primary motor cortex (but not the dorsolateral prefrontal cortex) as assessed via proton magnetic resonance spectroscopy. Glutamate-glutamine, myoinositol, and total choline levels were not significantly altered in either region. Brain structure: Heading had no significant effects on diffusion weighted imaging metrics. However, two blood biomarkers expressed in brain microstructures, glial fibrillary acidic protein and neurofilament light, were elevated 24 h (*p* = 0.014; g = 0.64) and ~ 7-days (*p* = 0.046; g = 1.19) post-Heading (*vs*. Kicking), respectively. Brain Function: Heading decreased tissue conductivity in 11 clusters located in the white matter of the frontal, occipital, temporal and parietal lobes, and cerebellum (*p*’s < 0.001) as assessed via electrical properties tomography. However, no significant differences were identified in: (1) connectivity within major brain networks as assessed via resting-state functional MRI; (2) cerebral blood flow as assessed via pseudo continuous arterial spin labelling; (3) activity within electroencephalography frequencies (infra-slow [0.03–0.06 Hz], theta [4–8 Hz], alpha [9–12 Hz], or beta [13–25 Hz]); or (4) cognitive (memory) function.

**Conclusions:**

This study identified chemical, microstructural and functional brain alterations in response to an acute non-concussive soccer heading task. These alterations appear to be subtle, with some only detected in specific regions, and no corresponding cognitive deficits observed. Nevertheless, our findings suggest that individuals should exercise caution when performing repeated non-concussive head impacts in sport.

*Trial registration* ACTRN12621001355864. Date of registration: 7/10/2021. URL: https://www.anzctr.org.au/Trial/Registration/TrialReview.aspx?id=382590&isReview=true.

**Supplementary Information:**

The online version contains supplementary material available at 10.1186/s40798-025-00867-0.

## Key Points

- In this randomised, controlled, crossover pilot trial of 15 male soccer players, a controlled non-concussive soccer heading task was found to alter select markers of brain function, chemistry and microstructure.
- These markers included brain tissue conductivity, total N-acetylaspartate and total creatine levels in the primary motor cortex, and plasma glial fibrillary acidic protein and neurofilament light concentrations. No corresponding cognitive deficits (and few adverse symptoms) were observed .
- These findings illustrate the importance of exercising caution when performing repetitive head impacts in sport.

**Supplementary Information:**

The online version contains supplementary material available at 10.1186/s40798-025-00867-0.

## Background

Athletes participating in contact/collision sports such as American football, rugby and association football (soccer) can sustain hundreds of head impacts each sporting season [1]. High strength, unanticipated and/or poorly located head impacts can cause *concussion*, a type of mild traumatic brain injury (mTBI) that is accompanied by a host of clinical symptoms (e.g., headache, blurred vision, nausea) [2]. Head impacts that do not elicit clinical symptoms are termed ‘*subconcussive*’, or increasingly ‘*non-concussive’* [1, 3]. These impacts are exceedingly common, accounting for > 99% of those incurred in sport [4, 5]. Some evidence also suggests that repetitive non-concussive impacts have the potential to cause long-term harm (e.g., neurodegenerative diseases), after years of exposure [6, 7]. Despite this, the effects of non-concussive impacts on the brain remain poorly understood [8].

Several observational studies have investigated the microstructural, chemical and functional effects of non-concussive impacts in sport; specifically, those incurred over the course of a sporting season [9–12]. These studies have employed various assessment techniques, including *central methods* (e.g., magnetic resonance imaging [MRI] sequences such as diffusion weighted imaging [DWI], magnetic resonance spectroscopy [MRS] and functional MRI [fMRI]), *peripheral methods* (e.g., blood biomarkers), and *functional tests* (e.g., cognitive tasks) [8, 13–15]. Recent systematic reviews summarising their findings indicate that non-concussive impacts have the potential to alter brain microstructure, chemistry and function [8, 13–15]. However, results are inconsistent [8, 13–15]. It should also be noted that observational studies have inherent limitations (e.g., confounders, biases) and cannot establish causation.

Interventional studies (particularly, randomised controlled trials [RCTs]) are increasingly being used to investigate the effects of non-concussive impacts on the brain [16]. When using these designs, impacts are typically administered in the form of a controlled non-concussive soccer heading task (SHT) [16]. Studies employing SHTs have shown that non-concussive impacts can elicit significant alterations in neuroelectric (via electroencephalography [EEG]) [17], cognitive [18, 19], neurovascular [20], neuroophthalmologic [21, 22], and vestibular [23, 24], function in healthy soccer players. SHTs have also been reported to increase blood concentrations of neurofilament light (Nf-L; a biomarker of axonal pathology) [25, 26], glial fibrillary acidic protein (GFAP; a biomarker of astroglial pathology) [27], and certain inflammatory markers [28]. However, to our knowledge no interventional studies have investigated the microstructural, chemical or functional effects of non-concussive impacts in sport using MRI techniques. This is important, as MRI has the capacity to interrogate regional chemical, functional and microstructural parameters that have not previously been investigated and allow researchers to predict the functional consequences of any changes observed.

The primary aim of this study was to investigate the acute effects of non-concussive impacts, administered in the form of a controlled SHT, on brain microstructure, function and chemistry using MRI; specifically, DWI, electrical properties tomography [EPT], blood-oxygen-level-dependent [BOLD] resting-state fMRI [rs-fMRI], pseudo continuous arterial spin labelling [pCASL]) and proton MRS [^1^H-MRS]). It was hypothesised that non-concussive impacts would alter these parameters, as per previous observational studies [8, 13, 15]. The secondary aim was to investigate the effects of non-concussive impacts on neuroelectric function (via EEG), cognitive function and blood biomarkers of axonal and astroglial damage (i.e., Nf-L, GFAP) and inflammation (e.g., interleukin [IL]-6, etc.).

## Methods

### Study Design

A randomised, controlled, crossover pilot trial was conducted at Neuroscience Research Australia (NeuRA; Randwick, NSW). The trial was approved by the University of Sydney’s Human Research Ethics Committee (2021/515), registered prospectively with the Australian New Zealand Clinical Trials Registry (ACTRN12621001355864) and conducted in accordance with the standards of ethics outlined in Declaration of Helsinki.

### Participants

Healthy individuals aged between 18 and 35 years and with ≥ 5 years of soccer heading experience were recruited via word-of-mouth and using a general advertisement (flyer) that was distributed to soccer clubs throughout Sydney (NSW, Australia). The full eligibility criteria are presented in Supplementary File S1. Briefly, the key exclusion criteria were: (1) a head, neck, face or eye injury (including a confirmed or suspected concussion) within the last 12 months; (2) an uncontrolled physical or mental health condition; (3) a neurological disorder; (4) a contraindication to MRI; and (5) pregnant or lactating. All participants were recompensed $200 for their time.

### Enrolment

Each volunteer completed a short online questionnaire and a telephone interview. Those suitable were then scheduled for a face-to-face screen with the trial coordinator (N.D.) and physician (K.R.). Here, they were informed about the nature and risks of the experimental procedures, before providing written informed consent and being assessed for eligibility. Eligible participants then provided demographic information (including an indication of ‘usual’ [non-specific] concussion symptoms as per the Concussion Recognition Tool [CRT]-5) [29] and practised the cognitive function tasks they would complete at their test sessions.

### Randomisation and Allocation Concealment

Participants were randomised to one of two possible condition orders in a 1:1 ratio at the beginning of their first test session. Specifically, they were assigned a unique identification code by the trial coordinator (N.D.) that was linked to a condition order via a pre-populated randomisation schedule. The schedule was generated in a series of balanced blocks (and one ‘block’ of one) by an investigator (E.C.) using an online random number generator (www.sealedenvelope.com; *Sealed Envelope Ltd, London, England*). The schedule could only be accessed by the investigator and one other researcher (P.A.), neither of whom had contact with participants. The balanced blocks also varied in size such that the final condition order within each block could not be predicted. Condition allocation was then concealed using sealed, opaque envelopes.

### Conditions

Conditions were administered by the trial coordinator (N.D.) and a second investigator (D.M.) on the outdoor fields of Paine Reserve (Randwick, NSW; ~ 500 m from NeuRA).

#### Intervention (‘Heading’)

The intervention was a SHT (‘Heading’). A JUGS Soccer Machine™ (JUGS® Australia, Cheltenham, Victoria, Australia) was used to launch FIFA regulation size 5 soccer balls at a speed of 35 mph (~ 56 km/h). Participants performed 20 headers in 20 min from a distance of ~ 12 m to the JUGS. They were instructed to hit the ball with their forehead and to direct it back towards the JUGS. Unsuccessful headers (i.e., where there was no observable contact between the head and the ball) were re-administered. This protocol was designed to be a realistic but demanding task for soccer players (i.e., of an intensity at the upper end of the normal range experienced during matches/training [30, 31]).

#### Control (‘Kicking’)

The control was a soccer kicking task (‘Kicking’). It was administered exactly as the intervention, except participants kicked (rather than headed) the ball (which was launched along the ground).

### Test Sessions

Participants completed two test sessions, Heading and Kicking, separated by a washout period of ≥ 7 days.

#### Standardisation Procedures

Prior to each session, participants were instructed to: (1) avoid soccer heading and playing other contact sports (> 7 days); (2) avoid using alcohol (> 24 h), caffeine (> 12 h), anti-inflammatory medication (> 4 days) and central nervous system (CNS) active drugs (> 7 days); (3) avoid moderate to strenuous exercise (> 12 h); (4) spend > 8 h in bed overnight; (5) consume a standardised breakfast (at home) and (6) consume 500 mL of water before arriving at the clinic.

#### Experimental Procedures

**Fig. 1:**
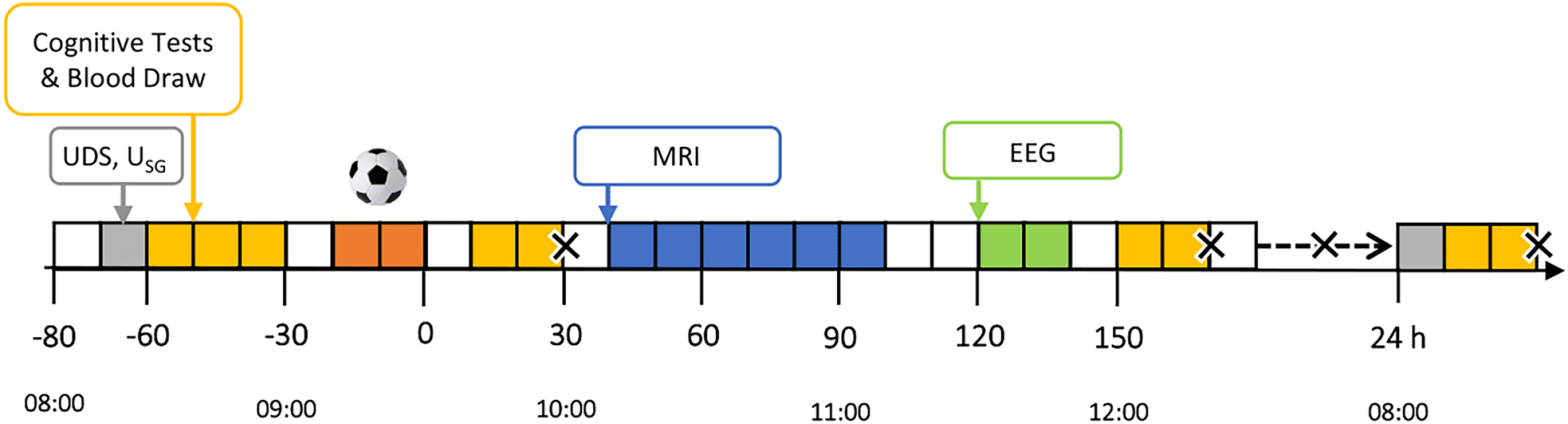
Experimental procedures. Grey represents UDS and U_SG_ checks; Yellow represents the cognitive tests and blood draws; Orange represents the trial intervention (SHT); Blue represents the MRI; Green represents the EEG. Crosses (X) are used to signify adverse event (concussion symptom) checks. Abbreviations: EEG–electroencephalography; MRI–magnetic resonance imaging; SHT: soccer heading task; UDS–urine drug screen; U_SG_–urine specific gravity.

The experimental procedures are summarised in Fig. 1. Briefly, participants arrived at NeuRA between ~ 7:30–8:30 AM and verbally acknowledged compliance with the standardisation procedures. A urine sample was collected to confirm avoidance of CNS active drugs (DrugCheck® NxStep Onsite, Care Health America, Blue Earth, USA) and to assess hydration status (urine specific gravity [U_SG_]; Palette Digital Refractometer, ATAGO, Bellevue, USA). If U_SG_ was > 1.024, likely indicating hypohydration [32], participants consumed 500 mL of water [33]. Individuals then completed a series of baseline assessments (‘Pre’; Sect. "Data Collection"), before they were walked to the outdoor field to receive their assigned condition (i.e., Heading or Kicking). Participants then returned to NeuRA to complete a series of post-condition assessments (‘Post’ and ‘2.5 h Post’). They left between 12:30 and 1:30 PM but returned the following day to complete their 24-h post-condition assessments (’24 h Post’). Participants were instructed to adhere to the same standardisation procedures prior to this visit.

### Data Collection

#### MRI Acquisition (Primary Outcome)

MRI scanning commenced ~ 45 min post-condition and took ~ 60 min to complete. The timing of this assessment was selected with consideration for pragmatic factors (e.g., participant transportation) and prior research suggesting that SHTs can elicit immediate alterations in neurovascular and corticomotor function [18, 20]. All images were collected by a registered radiographer using a 3T MRI scanner (Ingenia CX, Philips) with a 32-channel head coil. Participants were placed into the MRI scanner in a supine position with their head secured in a tight-fitting head coil with headphones to prevent movement. Images were collected in the following order (time of acquisition post-condition reported as mean ± SD): (1) T_1_-weighted anatomical (+ 48 ± 6 min); (2) ^1^H-MRS (+ 58 ± 7 min); (3) EPT (+ 74 ± 7 min); (4) BOLD rs-fMRI (+ 82 ± 12 min); (5) pCASL (+ 91 ± 8 min); and (5) DWI (+ 97 ± 8 min). Scans were conducted to measure brain chemistry (^1^H-MRS), function (EPT, rs-fMRI and pCASL) and microstructure (DWI). Participants were instructed to remain awake and focused on a crosshair (displayed on a screen) throughout functional scans.

*T1-weighted anatomical*: A high-resolution 3-dimensional anatomical image set covering the entire brain was acquired for accurate image registration and segmentation (211 sagittal slices; repetition time [TR]/echo time [TE] = 7.3/3.4 ms; flip angle = 8°; slice thickness = 0.9 mm; voxel size = 0.75 × 0.75x0.9 mm).

^*1*^*H-MRS*: Single voxel ^1^H-MRS was collected from two brain regions: the left dorsolateral prefrontal cortex (dlPFC) and primary motor cortex (M1) in the somatotopic region representing the dominant foot, as these regions have demonstrated neurometabolic alterations in previous observational studies of non-concussive impacts [34–36]. Data from ^1^H-MRS were collected using a semiadiabatic Localization by Adiabatic SElective Refocusing (sLASER) sequence (VAriable Power and Optimized Relaxations [VAPOR] water suppression; 64 averages; 2048 data points; TE = 31 ms for dlPFC and 33 ms for M1, TR = 5000 ms; voxel size = 15 mm^3^). Second order shimming was conducted using the auto-shimming function with the vendor-supplied (Phillips) sLASER sequence; only spectra with full width at half maximum (FWHM) values less than 15 Hz were accepted (otherwise scans were repeated).

*EPT*: Scans were acquired using a balanced fast field echo (bFFE) sequence (TR/TE = 2.54/1.27 ms; flip angle = 25°; nonselective radiofrequency [RF] pulses; compressed SENSE factor 1; RF shimming calibrated with full coverage 2D dual refocusing echo acquisition mode [DREAM]; voxel size = 1 mm^3^).

*rs-fMRI*: A rs-fMRI series consisting of 250 whole brain BOLD fMRI image volumes was collected (TR/TE = 1500/30 ms; 75 axial slices; voxel size = 2 mm^3^).

*pCASL*: A resting pCASL series covering the entire brain was acquired (TR/TE = 4188/10.7 ms; 24 axial slices; voxel size = 3 × 3x6 mm; 384 images). Four background suppression pulses were applied to maximise the sensitivity to blood perfusion [37].

*DWI*: A DWI set covering the entire brain was acquired using a single-shot multi-section spin-echo echo-planar pulse sequence (TR/TE = 3000/75 ms; flip angle = 90°; 57 axial slices; voxel size = 2.5 mm^3^). For each slice, diffusion gradients were applied along 32 phase-encoding directions at b-value = 1000 s/mm^2^, 64 phase-encoding directions at b-value = 3000 s/mm^2^, and one volume acquired at b-value = 0 s/mm^2^. Anatomical and diffusion image sets were visually inspected for artifacts; no participants were excluded from the analysis.

#### EEG Acquisition

A 15-min resting EEG recording was acquired ~ 2 h post-condition using a 64‐channel EEG system (ANT Neuro, Hengelo, Netherlands). Electrodes were placed according to the standard 10–20 system [38], with reference electrodes placed on opposing mastoid processes, and an electrode placed on the orbicularis oculi muscle to monitor eye movements. Participants were tested while seated in a quiet room and instructed to relax, close their eyes and let their mind wander. Continuous EEG data were acquired at a sampling rate of 1000 Hz with online band-pass filtered between 0.01 and 100 Hz.

#### Blood Acquisition

Blood was collected into 6.0 mL pre-treated EDTA vacutainers and 3.5 mL serum vacutainers at Pre, Post, 2.5 h Post and 24 h Post. Each vacutainer was centrifuged for 15 min at 1500 g and 4 ℃ within 30 min of collection (following coagulation of the serum sample), with plasma and serum stored at − 80 ℃ until analysis.

#### Cognitive Function Acquisition

Cognitive function was assessed at Pre, Post, 2.5 h Post and 24 h Post using two computerised tasks from the Cambridge Neuropsychological Test Automated Battery (CANTAB): the Paired Associate Learning (PAL; ~ 8 min duration) and Spatial Working Memory (SWM; ~ 4 min duration) tasks [39, 40]. These tasks have demonstrated sensitivity to the effects of SHTs [18]. Participants completed the tasks in a quiet room and were instructed to take their time and minimise errors.

### Data Processing and Analysis

#### MRI Processing and Analysis

^*1*^*H-MRS*: All analysis specifics, including visualisation of voxel placement and sample spectra, are presented in Supplementary File S2 (MRSinMRS Acquisition and Analysis Checklist) [41]. The spectrum and unsuppressed water spectrum for each participant were analysed by N.D. (unblinded) using Totally Automatic Robust Quantitation in NMR (TARQUIN; v4.3.10) [42]. Pre-processing consisted of eddy current correction, lipid filtering, automatic referencing water residual removal using Hankel singular value decomposition, zero-order phase correction, and automatic referencing using zero filling. The neurometabolites of interest were: total *N*-acetylaspatate (tNAA; NAA + *N* acetyl glutamate), myo-inositol (mI), total choline (tCho; choline-containing compounds), total creatine (tCr; creatine + phosphocreatine), and glutamate/glutamine (Glx). Neurometabolites were analysed and reported as water-referenced values (using the default TARQUIN processing) rather than using internal neurometabolite references (e.g., tCr), as several neurometabolites including tCr may be influenced by non-concussive impacts [15].

The quality of all spectra were examined using the line width/FWHM of fitted spectra and signal-to-noise ratio (SNR) using TARQUIN’s default processing. Data were excluded if FWHM > 15 Hz or SNR was < 5 (no data were discarded on this basis; Supplementary File S2). In addition, the accuracy of voxel placement was visually inspected through heat maps. Data from poorly placed voxels were discarded. Tissue parcellation (grey and white matter) within each voxel was reported (Supplementary File S2).

*EPT*: Data were processed by J.C. (blinded to condition) to produce conductivity maps according to methods described by Cao and colleagues [43]. In brief, T1-weighted turbo field echo images were co-registered and segmented into white matter, grey matter and cerebrospinal fluid using FSL [44], to alleviate boundary artifacts. Within each tissue type, an average parabolic phase fitting method was used to reduce artifacts amplified in the Laplacian [45], and the second derivatives of the fitted phase were taken to calculate conductivity. The conductivity maps of each participant from both sessions were normalised into Montreal Neurological Institute (MNI) space (voxel size 2 mm isotropic) using statistical parametric mapping (SPM) 12 [46].

*rs-fMRI*: Using SPM 12 [46], and custom software, fMRI images were processed by N.D. (unblinded). Images were slice-time and motion corrected, and global signal drifts removed using the detrending method described by Macey and colleagues [47]. Physiological noise was corrected (cardiac frequency band 60–120 beats per minute + 1 harmonic; respiratory frequency band 8–25 breaths per minute + 1 harmonic) using the DRIFTER toolbox [48], and the six-parameter movement-related signal changes modelled and removed using a linear modelling of realignment parameters procedure [47]. The fMRI images were then co-registered to each participant’s T1 anatomical image, the T1 image then spatially normalised to the MNI template and normalisation parameters applied to the fMRI images. The fMRI images were then spatially smoothed using a 6 mm FWHM Gaussian filter. Independent components analysis (ICA) was performed using the Group ICA toolbox [49] to define major brain networks [50–52]. Thirty independent components were extracted using the Infomax ICA algorithm [53], and major networks identified by visual inspection. We selected nine components from six major brain networks: the salience, sensorimotor, visual, default mode, cerebellar and executive control networks (Supplementary File S3).

*pCASL*: Using SPM 12 [46], pCASL data were analysed by N.D. (unblinded). All pCASL sets were realigned, co-registered to each participant’s source image, and a mean cerebral blood flow (CBF) map created using the subtraction method from the ASL toolbox [54]. Each participant’s source images were spatially normalised to MNI space and the parameters applied to the CBF maps. The CBF maps were smoothed using a 6 mm FWHM Gaussian filter.

*DWI*: During acquisition, a coding error occurred that corrupted the acquisitions with diffusion gradients at b-value = 1000 s/mm^2^. Consequently, b-1000 DWI were removed from the image set. Using SPM12 [46], the remaining images were processed by N.J. (blinded to condition). Images were corrected for motion, eddy current and b0 distortion. Elements of the diffusion tensor were computed from the images using a linear model, then fractional anisotropy (FA) and mean diffusivity (MD) whole-brain maps were derived. The FA and MD maps were resliced into 1.5 mm isotropic voxel sizes and co-registered to each individual’s T1-weighted anatomical image to ensure all images were in the same three-dimensional space. Subsequently, they were spatially normalised to MNI space using the previously calculated parameters from T1 images and spatially smoothed using a 5 mm FWHM Gaussian filter.

In addition, a fixel-based analysis (FBA) was conducted using MRtrix3 [55], to determine tract-specific quantities of fibre density (FD), fibre cross section (FC) and a combination of both (FDC). Data were processed by M.G. (blinded to condition) according to previous published methods [56].

#### EEG Processing and Analysis

Processing of EEG data were performed in Matlab (Version R2020b; MathWorks, Inc., Natick, MA, USA) and the FieldTrip toolbox by N.D. (unblinded) [57]. Prior to processing, data were bandpass filtered between 0.01 and 35 Hz. Initially, large artefacts and poor-quality channels were identified via visual inspection and removed from the data. Following this, an ICA was conducted to remove typical eye artefacts (e.g., blinks and saccades). Poor quality channels were reconstructed via interpolation from neighbouring channels. Finally, the EEG signals were re-referenced to the average of the mastoid electrodes and down sampled to 200 Hz to enhance processing speed. Estimates of cortical power were produced using the fast Fourier transform, at the following frequencies: 0.02–0.09 (at steps of 0.01 Hz), 0.1–0.9 (at steps of 0.1 Hz), and 1–30 (at steps of 1 Hz). The cortical power at each frequency was computed by averaging the power across all EEG channels. Four frequency bands were included for analysis: infra-slow (0.03–0.06 Hz), theta (4–8 Hz), alpha (9–12 Hz), and beta (13–25 Hz).

#### Blood Biomarkers Analysis

*Nf-L and GFAP*: Plasma samples were analysed in duplicate by a scientist (W.O.) blinded to condition using a Simoa HD-X Analyzer (Quanterix, Lexington, USA) and commercially available Simoa kits as per manufacturer’s instructions [58]. GFAP Discovery assays (Item 102,336) were used to quantify GFAP, with participant samples analysed on the same plate, and all samples measuring above the lower limit of quantification (LLOQ; 0.686 pg/mL). NF-Light V2 Advantage assays (Item 104,073) were used to quantify Nf-L, with all Pre and 24 h Post samples from the same participant analysed on the same plate, and the remaining samples analysed separately later (once additional funding was sourced). All samples measured above the LLOQ for Nf-L (1.38 pg/mL).

*Inflammatory Markers*: Serum samples were analysed by a Contract Research Organisation (Eve Technologies, Calgary, Canada). The Human Cytokine 15-Plex Assay Array was performed to determine concentrations of: granulocyte–macrophage colony-stimulating factor, interferon gamma, interleukin (IL)-1β, IL-1RA, IL-2, IL-4, IL-5, IL-6, IL-8, IL-10, IL-12p40, IL-12p70, IL-13, monocyte chemoattractant protein-1 (MCP-1), and tumour necrosis factor-α. The analyses were performed in duplicate by a laboratory technician blinded to condition. Only Pre and 24 h Post samples were analysed due to funding constraints.

#### Cognitive Function Outcomes

The PAL task measures visual memory and learning [39]. The outcome measures were: number of attempts required to complete the task (‘attempts’) and number of errors made (adjusted for attempts if the participant did not complete the task; ‘adjusted errors’). The SWM task measures working memory, executive functions, and strategy [40]. The outcome measures were: number of errors (‘errors’) and a strategy score (calculated based on the randomness of participants’ opening boxes, where lower scores indicated better strategy; ‘strategy’).

#### Condition Characteristics

An impact monitoring mouthguard (Prevent Biometrics™, Edina, MN, USA) was used to measure linear and rotational acceleration of head impacts (peak linear acceleration [PLA]; peak rotational acceleration [PRA]). This device has demonstrated a high degree of accuracy in controlled and field environments (concordance correlation coefficient > 0.8) [59]. Participants were also asked to rate how ‘well’ they performed each header on an 11-point scale (− 5 = ‘very poorly’; to + 5 = ‘very well’) and the ‘strength’ of each header on a 5-point scale (1 = ‘very low’; to 5 = ‘very high’). Mean heart rate (HR) throughout the 20-min activity was also determined using a chest strap monitor (Polar H10 HR Sensor).

#### Adverse Event Monitoring

Participants were monitored for signs of concussion (adverse event [AE]) using Parts 1–3 of the CRT-5 [29], at Post, 2.5 h Post, 4–8 h Post and 24 h Post Task (Fig. 1). Parts 1 and 2 were used to identify ‘red flag’ and ‘observable sign(s)’ of concussion. Part 3 was used to identify possible (non-specific) ‘symptoms’ of concussion. Participants answered ‘yes’, ‘no’ or ‘maybe’ to the ‘red flag(s)’, ‘observable sign(s)’ and potential ‘symptoms’. Responses were documented, reviewed and escalated to the trial physician, as necessary.

### Sample Size

A target sample size of 15 was selected based on practical considerations such as time, cost, and resource allocation, rather than an a priori power calculation. However, a sensitivity power calculation was performed [60]. Using a two-sided α of 0.05 and a sample size of 15, we predicted our study would have 75% power to detect a difference between two dependent means of at least Cohen’s d_z_ = 0.73.

### Statistical Analyses

The EPT, fMRI, ASL and DWI data were analysed using Matlab (Version R2023b; MathWorks, Inc., Natick, MA, USA) and EEG data using Matlab (Version R2020b). The remaining data were analysed using R (Version 4.2.2) [61].

#### Electrical Properties Tomography (EPT), Resting-State Functional Magnetic Resonance Imaging (rs-fMRI), Arterial Spin Labelling (ASL) and Diffusion Weighted Imaging (DWI)

Second level, random effects, paired analyses were conducted to identify significant differences at a voxel-by-voxel level (*p* < 0.05, false discovery rate [FDR] corrected, minimum cluster = 10 contiguous voxels). For the rs-fMRI network analyses, each analysis was restricted by creating a mask of the relevant network using images from scan on both conditions (*p* < 0.05, FDR corrected). Significant differences for all MRI scans were then overlaid onto a mean T1-weighted anatomical image set.

#### Diffusion Weighted Imaging (DWI) Fixel-Based Analysis

A general linear model was fitted to every fixel to compare each metric (FD, FC, FDC) by condition. A whole brain tractogram consisting of two million streamlines was used for statistical inference using connectivity-based fixel enhancement [56]. Data were analysed by condition using non-parametric permutation testing (5000 permutations; *p* < 0.05, family wise-error [FWE] corrected).

#### Electroencephalography (EEG)

The global cortical power of each frequency band (i.e., infraslow, theta, alpha and beta) were compared by condition using paired t-tests with significance set a *p* < 0.05. In addition, to identify a group of channels where significant differences existed, the spatial distribution of power differences between conditions and within infraslow, theta, alpha and beta bands, were examined using cluster-based permutation tests (4000 permutations; *p* < 0.05, corrected using the ‘cluster’ function) [62].

#### Other Data

Continuous variables (e.g., neurometabolites [^1^H-MRS], blood biomarkers, condition characteristics) were analysed using linear mixed-effects models and the ‘lme4’ and ‘emmeans’ packages [63, 64]. The ‘single-point’ models included Condition as a fixed effect with Participant as a random effect. The ‘serial’ models included Condition, Time, and their interaction as fixed effects with random intercepts and slopes for Participants varying by Condition. The models were generated using the restricted maximum likelihood criterion and no covariance structure was specified (unstructured). If the residuals were non-normally distributed (Shapiro–Wilk test, *p* < 0.05) or heteroskedastic (Levene test, *p* < 0.05), the data were square-root transformed and reanalysed (and if unimproved, log-transformed). If an appropriate model could not be generated, the ‘best’ of those described above (i.e., simplest model violating the fewest assumptions) was utilised. Note: As plasma Nf-L concentrations can remain elevated for 22 days following SHTs [25], an additional analysis was conducted, using Session (1 or 2), Condition Order (Heading–Kicking or Kicking–Heading) and their interaction as a fixed effects with random intercepts and slopes for Participant varying by Condition Order.

Count variables (e.g., cognitive function metrics) were analysed using generalised linear mixed-effects models and the ‘glmmTMB’ and ‘emmeans’ packages [64, 65]. These models included the same fixed and random effects as those specified above, but were fitted to a Poisson distribution, unless over-dispersed, and/or zero-inflated. In these instances, a negative binomial, zero-inflated Poisson, or zero-inflated negative binomial distribution was substituted, respectively (with both parts of the zero-inflated models containing the same fixed effects).

Two-sided (Dunn–Šidák corrected) pairwise comparisons were used to compare estimated marginal means across Condition if a significant main effect of Condition, or a Condition $\times$ Time interaction (or equivalent for Nf-L) was observed. Normally distributed data were presented as Mean ± SD and non-normally distributed data were presented as Median [IQR]. Statistical significance was accepted as *p* < 0.05 and effect sizes were calculated as Hedges’ *g* [66].

## Results

### Participant and Condition Characteristics

**Fig. 2:**
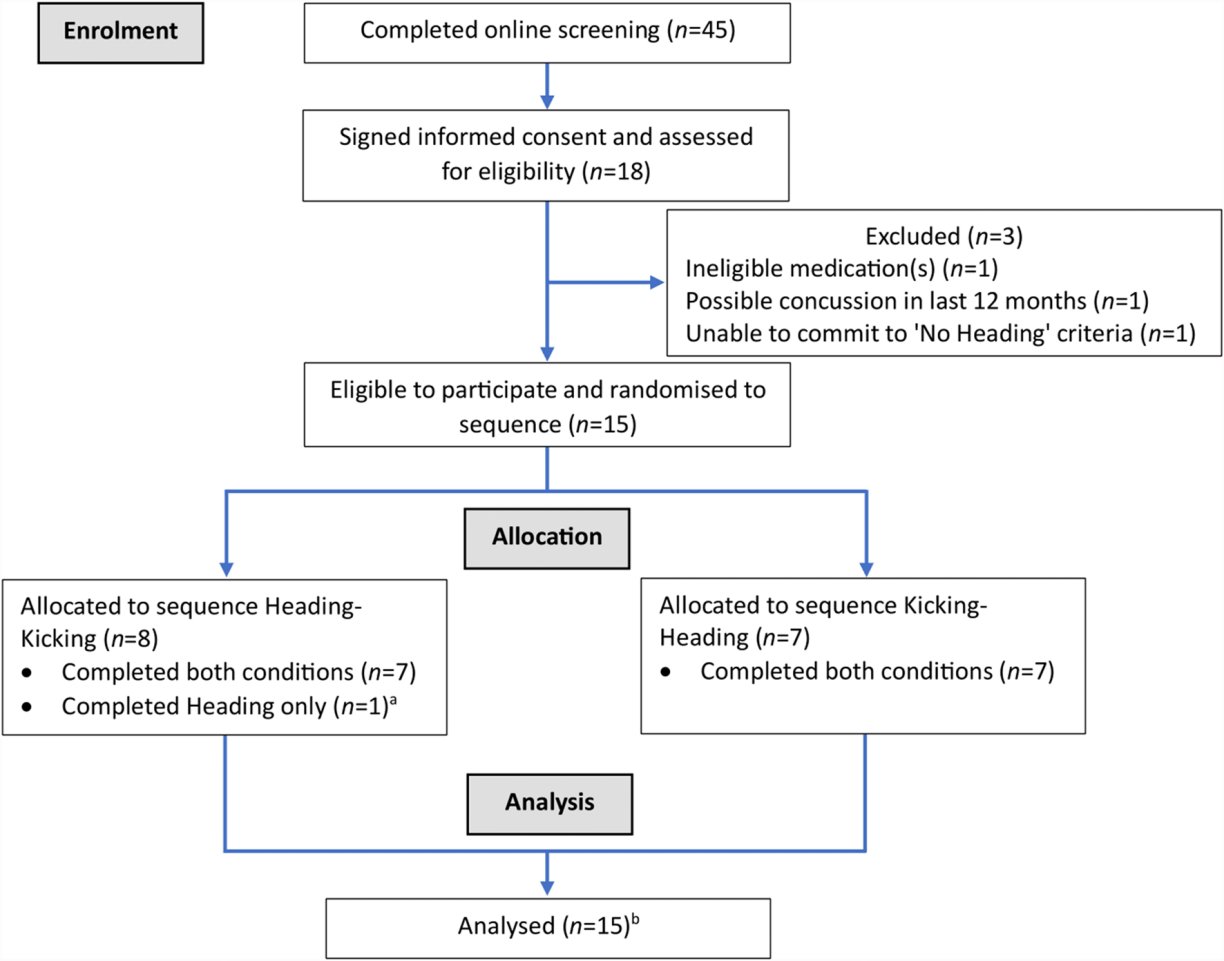
CONSORT Participant Flow Diagram. ^a^Did not complete the second test session (Kicking) for personal reasons. ^b^Except where the specific analytical technique could not handle missing data

Recruitment commenced in November 2021 and concluded 12 months later. Eighteen volunteers signed informed consent and 15 were randomised (Fig. 2). Of those randomised, 14 (93%) completed both conditions (i.e., as intended) and one completed one condition (i.e., Heading only) after being unable to complete the second test session for personal reasons. All 15 participants were included in the final sample (except where the analytical technique employed could not handle missing data) (Table 1). Participants completed their sessions between seven and 25 days apart (9 ± 4 days). Note that due to difficulties with recruitment (i.e. few females expressing interest in participation), only males completed this trial.

**Table 1:** Participant characteristics

ID	Age (years)	BMI (kg/m^2^)	Dominant foot	Predominant playing position	Headers in last 12 months^a^ (n)	Heading experience (years)	Previous concussions (n)	Time since last concussion (years)	Time of season^b^	Washout (days)
1	24	28.1	Right	Centre Midfield	528	18	0	NA	Pre-Season	25^c^
2	22	23.6	Right	Centre Attacking Midfield	928	14	0	NA	Pre-Season	7
3	25	24.2	Right	Centre Back	160	13	0	NA	In-Season	7
4	29	24.0	Right	Centre Defending Midfield	192	20	0	NA	In-Season	7
5	18	19.0	Right	Wide Back	336	9	0	NA	Off-Season	8
6	34	28.4	Right	Centre Defending Midfield	1520	28	2	17	In-Season	7
7	20	23.3	Right	Wing	144	8	1	8	In-Season	8
8	20	29.7	Right	Centre Back	96	8	2	4	In-Season	7
9	27	25.6	Right	Centre Back	1760	14	0	NA	Post-Season	8
10	22	24.0	Right	Centre Back	0	5	0	NA	Off-Season	8
11	32	28.2	Right	Centre Back	540	25	0	NA	Post-Season	7
12	20	25.1	Right	Striker	1248	8	0	NA	Post-Season	NA^d^
13	29	25.8	Left	Centre Back	832	23	2	5	Post-Season	8
14	27	24.3	Right	Centre Midfield	12	19	0	NA	Off-Season	8
15	27	24.3	Right	Centre Back	204	20	5	4	Off-Season	9
Mean ± SD	25 ± 5	25.2 ± 2.7			567 ± 549	16 ± 7				9 ± 4

Abbreviations: BMI–mass index

^a^The number of head impacts in the last 12 months was assessed via self-report using standardised questionnaire [30]

^b^Pre-Season: Scheduled training prior to In-Season but after Off-Season—possible infrequent games; In-Season: Scheduled training and frequent competitive games [at least weekly]; Post-Season: No/minimal scheduled training and between In-Season and Off-Season—possible infrequent games; Off-Season: No scheduled training/games

^c^Participant had longer than anticipated time between test session due to being diagnosed with SARS-CoV-2 between test sessions 1 and 2

^d^Participant did not complete test session 2 for personal reasons

Condition characteristics are summarised in Supplementary File S4. The average PLA and PRA of headers was 15.8 ± 5.6 g and 1271 ± 602 rad/s^2^, respectively. No head impacts were recorded on Kicking. Participants rated the strength of headers as 3 (IQR: 2–4) on a 5-point scale and how well they performed each header as 1 (IQR: − 1–3) on a − 5 to 5 scale. Mean HR tended to be higher during Heading than Kicking (81 ± 12 *vs.* 79 ± 12 bpm, *p* = 0.081, g = 0.17).

### Magnetic Resonance Imaging (MRI)

Only the 14 participants who completed both conditions could be included in the MRI analyses, except for the ^1^H-MRS analysis (details below).

#### Proton Magnetic Resonance Spectroscopy (^1^H-MRS)

Fifteen participants were included in the dlPFC analyses. Only 14 were included in the M1 analyses due to inaccurate voxel placement (on both sessions; ID: 1). Heading increased tNAA (*p* = 0.012, g = 0.66) and tCr (*p* = 0.010, g = 0.77) levels in the M1 compared to Kicking. No other significant differences were observed in either region (Table 2; all *p*’s > 0.05).

**Table 2:** Neurometabolite levels between trials assessed using proton magnetic resonance spectroscopy

ROI	Metabolite	Levels				*p* value	Hedges’ g
		Kicking		Heading			
dlPFC							
	Glx	7.04 ± 1.57		7.95 ± 1.76		0.156	0.55
	tNAA	9.04 ± 0.89		8.74 ± 0.76		0.333	− 0.35
	tCr	5.99 ± 0.89		5.95 ± 0.36		0.993	− 0.06
	tCho	1.74 ± 0.26		1.71 ± 0.17		0.659	− 0.12
	mI	3.12 ± 0.67		3.02 ± 0.65		0.619	− 0.15
M1							
	Glx	6.99 ± 1.01		7.55 ± 1.57		0.287	0.42
	tNAA	9.02 ± 0.50		9.43 ± 0.71		**0.012**	0.66
	tCr	5.53 ± 0.31		5.82 ± 0.44		**0.010**	0.77
	tCho	1.56 ± 0.15		1.56 ± 0.12		0.894	− 0.04
	mI	2.88 ± 0.54		2.76 ± 0.36		0.511	− 0.26

Abbreviations: dlPFC – dorsolateral prefrontal cortex, Glx – glutamate and glutamine, M1 – primary motor cortex, mI – myo-inositol, mM (millimolar), tNAA – total *N*-acetyl aspartate (*N*-acetyl aspartate and *N*-acetyl glutamate), ROI–region of interest, tCho–total choline (choline-containing compounds), tCr–total creatine (creatine and phosphocreatine). Data presented as mean ± SD. **Bold** values represent statistically significant changes (*p* < 0.05)

#### Electrical Properties Tomography (EPT)

**Fig. 3:**
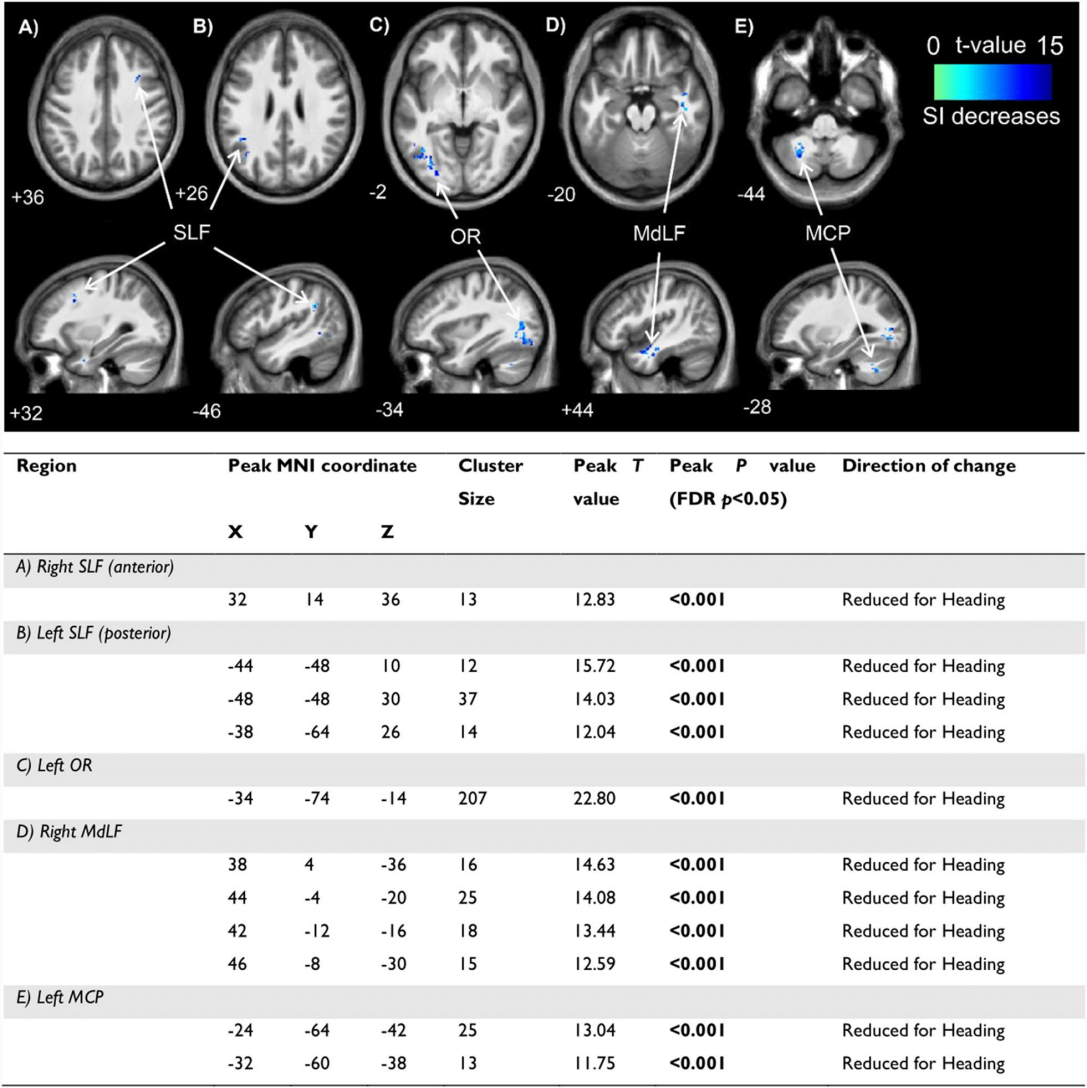
Locations of significant clusters from electrical properties tomography data (*above*) and statistical details (*below*) between conditions in **A** right SLF (anterior); **B** left SLF (posterior); **C** left OR; **D** right MdLF; and **E** left MCP. Cooler (blue) colours represent higher t values and a decrease in signal intensity. Location of each sagittal and axial slice in Montreal Neurological Institute space are indicated at the bottom left of each slice. Abbreviations: MCP–middle cerebellar peduncle; MdLF–middle longitudinal fasciculus; MNI–Montreal Neurological Institute; OR–optic radiation; SI–signal intensity; SLF–superior longitudinal fasciculus

Heading significantly reduced tissue conductivity in 11 clusters located in the white matter of the frontal, occipital, temporal and parietal lobes, and cerebellum. The location, size and *T* values of these clusters are presented in Fig. 3. No significant increases in conductivity were observed.

#### Resting-State Functional Magnetic Resonance Imaging (rs-fMRI)

Heading had no significant effects on network connectivity strength in any of the six brain networks identified (i.e., the salience, sensorimotor, visual, default mode, cerebellar and executive control).

#### Pseudo Continuous Arterial Spin Labelling (pCASL)

Heading had no significant effects on resting CBF in any brain region.

#### Diffusion Weighted Imaging (DWI)

Heading had no significant effects on FA or MD in any brain region. In the fixel-based analysis, Heading had no significant effects on FB, FC or FBC in any brain region (example images from the analysis provided in Supplementary File S5).

### Electroencephalography (EEG)

Of the 14 participants who completed both conditions, 12 were included in these analyses. Indeed, EEG data could not be collected for one participant (on Kicking) due to technical difficulties and was unreadable for another (on both conditions).

**Fig. 4:**
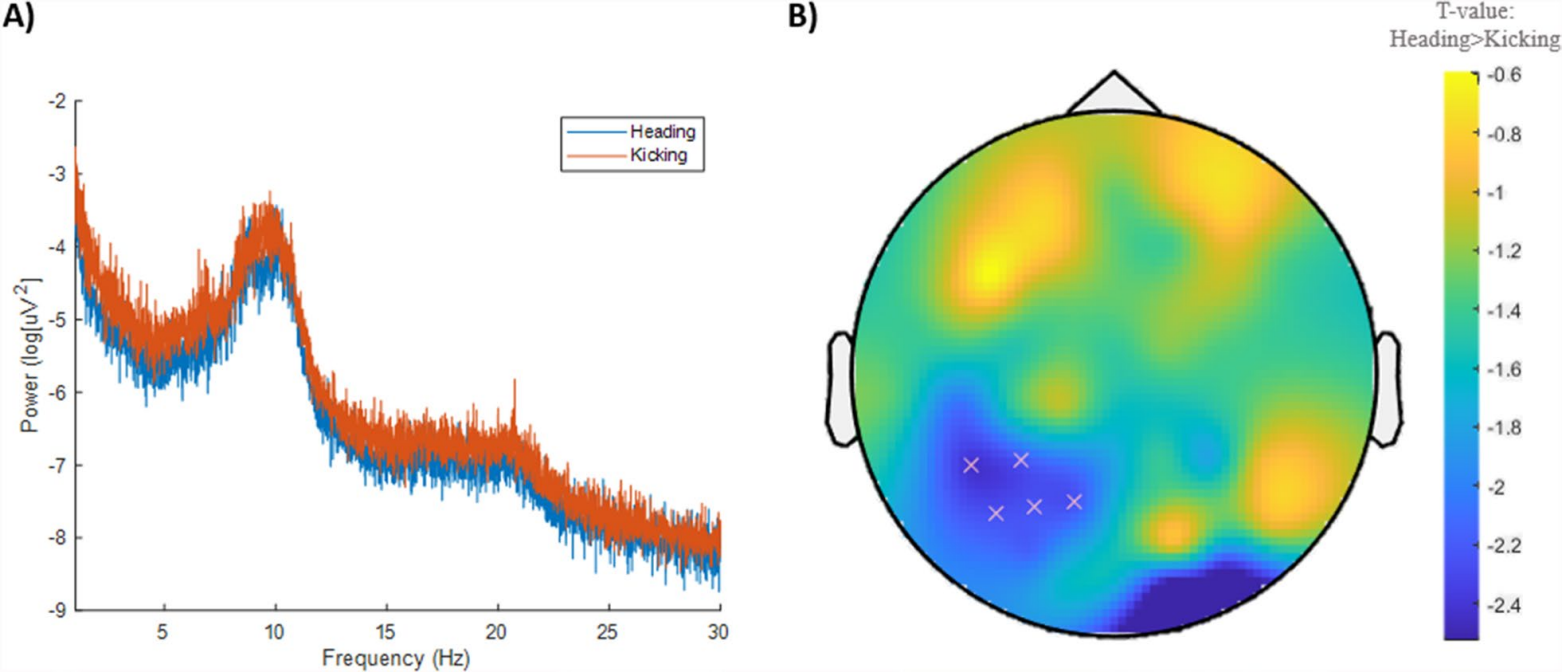
Electroencephalography data between conditions. **A** Power spectra across all frequencies between conditions. **B** A trend (*p* = 0.066) for reduced alpha frequency power in five left posterior channels on Heading. Cooler (blue) colours represent higher t values and a decrease in power (scale provided on right)

Heading had no significant effects on infraslow, theta, alpha or beta frequency bands (Supplementary File S6). For cluster analyses, no significant differences for any individual band were identified (all *p* > 0.05, Fig. 4A). However, Heading tended to decrease alpha frequency power in five left posterior channels compared to Kicking (Fig. 4B; *p* = 0.066).

### Blood Biomarkers

#### Plasma Neurofilament-Light (Nf-L) and Glial Fibrillary Acidic Protein (GFAP)

Fifteen participants were included. Nine of the 120 samples (7.5%) could not be collected due to difficulties with vascular access, and four (3.3%) could not be analysed for one biomarker (specifically, Nf-L) due to limited sample volume.

**Fig. 5:**
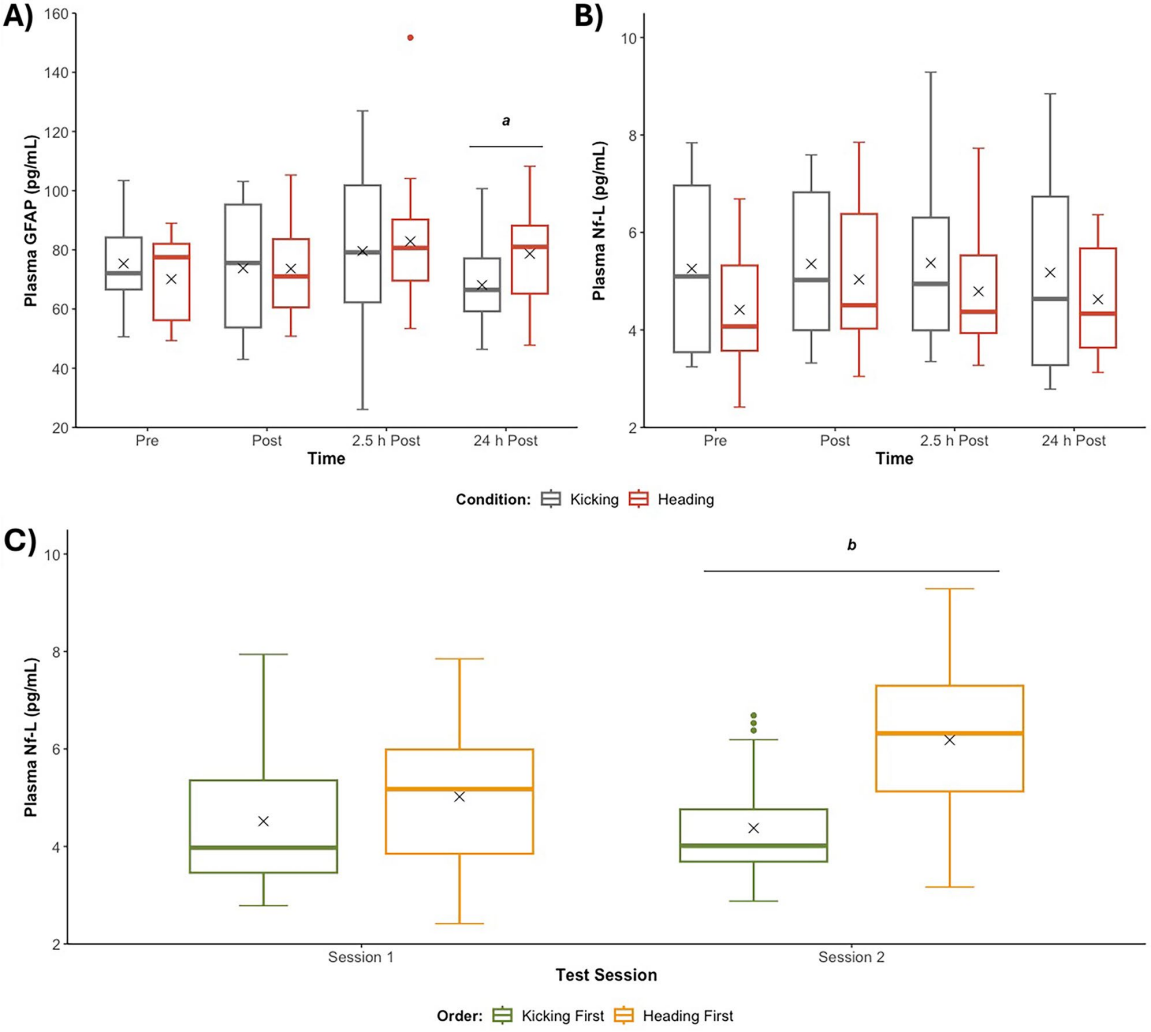
Plasma biomarker concentrations. **A** Plasma GFAP concentrations Pre, Post, 2.5 h Post and 24 h Post Heading vs. Kicking; **B** Plasma Nf-L concentrations Pre, Post, 2.5 h Post and 24 h Post Heading vs. Kicking; and **C** Plasma Nf-L concentrations on Session 1 and Session 2 when participants received Heading First vs. Kicking First. 'X' represents the mean value. Note that the best possible model violating the fewest assumptions (i.e., untransformed) was used for analysis of GFAP (Shapiro–Wilk, *p* > 0.05; Levene, *p* = 0.002). Abbreviations: GFAP–glial fibrillary acidic protein; mL–millilitre; Nf-L–neurofilament light; pg–picogram. ^a^Heading differs from Kicking (*p* < 0.05, g = 0.64), ^b^Heading First differs from Kicking First (*p* < 0.05, g = 1.19)

For Nf-L, the intra-assay coefficient of variation (CV) for the duplicate samples was 6.0 ± 5.1%. There was no significant interaction between Condition and Time (*p* = 0.510; Fig. 5A). However, a significant interaction between Session and Condition Order (*p* < 0.001) was observed (Fig. 5B). Post hoc comparisons showed that participants had higher plasma Nf-L concentrations on Session 2 with Condition Order Heading–Kicking (*n* = 8) than Kicking–Heading (*n* = 7) (6.60 [IQR: 4.64–7.63] vs. 3.70 [IQR: 3.47–4.82] pg/mL; *p* = 0.046; g = 1.19). No significant difference between Condition Order were observed at Session 1 (5.18 [IQR: 3.85–5.51] vs. 3.76 [IQR: 3.34–4.92] pg/mL; *p* = 0.765, g = 0.33).

For GFAP, the intra-assay CV for the duplicate samples was 5.5 ± 5.1%. A significant interaction between Condition and Time was observed (*p* = 0.043; Fig. 5C). Post hoc comparisons showed that Heading increased GFAP concentrations at 24 h Post compared to Kicking (81.0 [IQR: 65.2–88.2] vs. 66.5 [IQR: 59.3–77.1] pg/mL; *p* = 0.014; g = 0.64). No other significant differences were observed (all *p*’s > 0.05).

#### Serum Inflammatory Markers

Fifteen participants were included. Three of 60 samples (5%) could not be collected due to difficulties with vascular access. The intra-assay CV for the samples ranged from 11.8 ± 11.7% (MCP-1) to 36.0 ± 27.6% (IL-6). Due to extremely high CVs, the utility of data is limited. We have provided the results of these analyses in Supplementary File S7 for completeness, but recommend they be interpreted with caution.

### Cognitive Function

Fifteen participants were included. No significant Condition, Time or Condition $\times$ Time interactions were observed (all *p*’s > 0.05; Supplementary File S7). Note: SWM errors were not formally analysed as participants demonstrated a high degree of accuracy on all conditions and time points (i.e., achieved zero errors on 81% of occasions) (Supplementary File S7).

### Adverse Event (AE) Monitoring

The frequency of possible (but non-specific) symptoms of concussion are presented in Supplementary File S8. The most commonly reported symptom was ‘Pressure in the head’ (9/15; 60%) Post-Heading, followed by Headache (6/15; 40%) 2.5 h Post-Heading. Both abated within 24 h. The remaining symptoms were relatively infrequent. No participants experienced a concussion.

## Discussion

This RCT investigated the acute effects of non-concussive impacts on brain function, chemistry and microstructure utilising MRI and other techniques. Contrary to our hypothesis, significant changes were only observed in select outcomes. For brain function, reductions in tissue conductivity were found in several predominantly posterior white-matter brain regions. A trend toward reduced alpha frequency power (assessed via EEG) was also observed in the left parietal/occipital cortex. However, no changes in brain network connectivity (assessed via rs-fMRI) or CBF (assessed via pCASL) were found. Regarding brain chemistry, non-concussive impacts were found to increase tNAA and tCr in the M1 (but not the dlPFC), as assessed via ^1^H-MRS. Lastly, we found no significant effects of non-concussive impacts on brain microstructure, as assessed via DWI. However, two blood biomarkers expressed in brain microstructures, GFAP and Nf-L, were significantly elevated 24 h and ~ 7-days post Heading, respectively. These changes were observed in the absence of marked adverse symptoms and detectable alterations in cognitive function.

To our knowledge, no previous studies have used EPT to investigate the effects of non-concussive (or concussive) impacts on the electrical properties of the brain. The electrical properties of brain tissue, including conductivity and permittivity, are primarily dictated by intracellular and extracellular fluid volumes, ionic concentrations, and properties of cellular membranes (e.g., polarisation) within the tissue [67]. Our findings of reduced tissue conductivity in several predominantly posterior white-matter brain regions could, therefore, be indicative of changes to some of these biophysical properties. However, the precise etiology of this response is ambiguous, given that alterations to outcomes in other MRI scans (e.g., CBF via pCASL) were not found in these regions or were not measured (e.g., due to a priori selection in single voxel ^1^H-MRS based off previous literature [34–36]). Nonetheless, the dominant white matter response may be explained by its anisotropic and rheological properties, which make white matter more susceptible to alterations than grey matter [68–70]. In addition, the general location of alterations could represent a contrecoup mechanism, whereby the movement of the brain within the skull produces a secondary impact posteriorly [71].

The largest cluster demonstrating reduced tissue conductivity (> 200 voxels) was located posteriorly across the left optic radiation, which transmits visual information to the visual cortex [72]. Our participants did not report visual symptoms (e.g., double vision, blank or vacant look). However, previous interventional studies have shown that non-concussive impacts can affect oculomotor and neuro-ophthalmologic function [21, 22]. Interestingly, this general region of the brain also tended to demonstrate reduced alpha activity (as assessed via EEG), which could indicate altered excitability in visual regions or visual processing [73, 74]. That said, a previous interventional study utilising EEG found that whole brain and channel-wise alpha power was unchanged following a SHT when compared to the control condition [17]. Thus, the effect of non-concussive impacts on EEG metrics and in relation to visual function requires further investigation.

Other functional metrics including brain network connectivity (assessed via rs-fMRI), CBF (assessed via pCASL) and cognitive (memory) function, showed no significant effects of non-concussive impacts. Previous observational studies have detected increases in brain network connectivity (in sensorimotor, visual and cerebellum networks) [52] and CBF following non-concussive impacts [75, 76]. However, these studies used longer exposure periods (e.g., a full season) and different contact sports (e.g., American Football) which could potentially produce stronger effects. Observational studies also often have significant limitations (e.g., confounders, biases) that are difficult to control [77]. That said, one interventional study did find that non-concussive impacts altered performance on the same neurocognitive tasks used in this investigation [18]. Specifically, errors on both the SWM and PAL tasks were increased following heading, compared to baseline [18]. However, it also utilised a more demanding SHT (i.e., 20 headers in 10 min, at a speed of ~ 39 km/hr and distance of 6 m) [18]. Ultimately, with neither brain network connectivity nor CBF demonstrating significant alterations (including brain regions involved in memory [78]), it is not overly surprising that cognitive function was unaltered in the current study.

Two recent meta-analyses have investigated the effects of non-concussive impacts on brain chemistry as assessed via ^1^H-MRS [15, 79]. Both included observational studies only, as interventional studies were lacking. Neither meta-analysis identified significant differences in tNAA, tCr, tCho, and Glx levels between ‘cases’ (i.e., individuals exposed to non-concussive impacts) and controls [15, 79]. However, one found that tNAA (considered a biomarker of energy utilisation and neuronal health in mTBI [34, 80]) and tCr (considered a biomarker of energy homeostasis [80]) levels decreased from the pre-season to the mid-/post-season period [15]. In contrast, our study found that non-concussive impacts *increased* tNAA and tCr levels within the M1. This could be indicative of mitochondrial hypermetabolism in this region [80]. Again, the inconsistent findings could be due to methodological differences between studies. Alternatively, these studies may be detecting the same response along a temporal continuum (e.g., initial hypermetabolism as seen in concussive impacts [81], followed by delayed hypometabolism). Finally, the absence of frontal alterations (i.e., in the dlPFC) could be representative of a contrecoup mechanism as suggested earlier.

The current study used two analytical approaches to interrogate DWI data. No significant effects were observed on FA or MD, nor on the FBA metrics of FC, FD or FDC; suggesting that microstructural tissue alterations were not readily apparent [82]. Two recent systematic reviews have summarised the effects of non-concussive impacts on brain microstructure via DWI [13, 83]. Both reviews (which again included observational studies, only) concluded that non-concussive impacts typically, albeit somewhat inconsistently, decrease FA and increase MD in predominantly white matter regions [13, 83]. As previously noted, the inconsistent findings could be due to methodological differences between studies. To our knowledge, no studies have previously investigated the effects of non-concussive impacts on DWI using FBA.

While no significant microstructural alterations were observed using DWI, two blood biomarkers expressed in brain microstructures, Nf-L and GFAP, were significantly elevated post-Heading. First, a 1.78-fold increase was observed for the axonal injury marker, Nf-L, ~ 7-days post Heading. Three previous interventional studies have likewise shown that non-concussive impacts increase blood Nf-L concentrations and that concentrations can remain elevated for a prolonged period (e.g., up to 22 days) [25, 26, 84]. Though, in these studies, differences emerged within 24 h. This could be because a more demanding SHT was employed (e.g., one study administered 40 headers in 20 min at a speed of ~ 77 km/hr) [25]. Alternatively, we might have been unable to detect an effect at 24 h because of condition order effects. Nonetheless, the observed difference in plasma Nf-L concentration suggests some degree of axonal disruption [85]. The delayed response is consistent with several recent observational studies with serial Nf-L measures after concussive and non-concussive impacts in sport [86–88], but our concentrations remained well below those observed after diagnosed concussion (e.g., 6.6 *vs*. 10.0 pg/mL ~ 7 days post) [58]. In addition, the disparity between this finding and the results from our DWI analysis, may reflect a greater sensitivity of the blood biomarker to detect subtle microstructural alterations.

Second, a 1.22-fold increase in plasma concentrations of the astroglial pathology marker, GFAP, was observed 24 h post Heading. Two previous interventional studies have investigated the effects of non-concussive impacts on plasma GFAP concentrations [27, 84]. One found no difference 2 h or 24 h post Heading (compared to a Kicking control) [84], while the other found that concentrations were elevated 2 h, but not 24 h, post-Heading (compared to baseline) [27]. The significant and delayed response observed in our trial could be due to the fact that our SHT was more demanding than that utilised in previous investigations [27, 84]. Evidence suggests that 24 h may represent the peak for blood GFAP levels in mild TBI [89], and elevated levels at this time-point following non-concussive exposure are supported by recent findings from observational research in Australian football [88]. In any case, the observed change in plasma GFAP concentration suggests an astrocytic response, such as astrogliosis, or disruption to astrocyte integrity [27, 90]. Again, it should be noted that, although elevated, plasma GFAP concentrations remained below those previously observed after diagnosed concussion (e.g., 80 vs 100 pg/mL ~ 24 h post) [58]. This change was observed in the absence of changes to other markers of astrocyte activation/disruption including DWI metrics, infraslow oscillations (via EEG) or CBF (via pCASL) [91, 92].

Our study was not without limitations. First, it had a relatively small sample size; thus, may be underpowered to detect additional effects. A larger and more definitive trial is needed to verify the effects of non-concussive impacts on brain chemistry, function and microstructure in healthy male soccer players (and other populations). Second, we could not blind participants or researchers involved in trial activities to conditions. Third, we are unable to comment on the effect of the SHT on serum inflammatory markers due to extremely high CVs between duplicate samples. Fourth, while all participants spent at least 8 hours in bed, we did not monitor their sleep, and differences in the quality or duration could have influenced the results. Finally, as no previous interventional studies have investigated the effects of non-concussive impacts on brain structure, chemistry, and function using MRI techniques (and no ‘Pre’ or serial ‘Post’ scans were obtained), we cannot be sure how long the observed functional and chemical effects might persist nor certain that our 7-day washout period was sufficient to prevent carryover effects (i.e., in our MRI metrics). That said, it is reassuring to note that corticomotor inhibition [18], postural control [93], cognitive function [18], vestibular function [23], and concussion symptoms [94] have all been reported to normalise < 48 h post-SHT. In fact, to our knowledge, the only measure that has been reported to remain altered (elevated) ≥ 48 h post-SHT is plasma NF-L concentration [25], and these carryover effects were handled in our statistical analyses. We can also confirm that plasma GFAP concentration and our cognitive function metrics (which were measured ‘Pre’) did not demonstrate significant carryover effects (as illustrated in Supplementary File S9).

Our study only included young, healthy male soccer players with ≥ 5 years of soccer heading experience. Thus, the results may not be generalisable to other populations (e.g., females, inexperienced soccer players, clinical populations). For example, some observational research suggests that non-concussive impacts have different effects on brain structure [95] and chemistry [96] in female compared to male contact sport players. Head impact kinematics (e.g., linear acceleration, angular velocity) have also been reported to differ between males and females performing the same SHT (though few studies have compared the physiological effects of SHTs by sex, specifically) [97]. In addition, it should be noted that while some individuals may perform 20 headers in a game [30, 31] or training session (e.g., Table 1), others may not. Thus, results may not be generalisable to players with lower heading exposure. Soccer headers performed in game-based situations might also differ (e.g., biomechanically) from those performed under experimental conditions and, therefore, elicit different effects. Further research to determine whether minor symptoms such as ‘pressure in the head’ are indicative of physiological changes and to identify a potential threshold (e.g., change in tissue conductivity) at which these symptoms arise is also worthwhile.

## Conclusions

This RCT demonstrates that non-concussive impacts, specifically those administered in the form of a controlled SHT, can alter select markers of brain function, chemistry and microstructure in male soccer players. These include changes to brain tissue conductivity, tNAA and tCr levels in the M1, and plasma Nf-L and GFAP concentrations. These alterations appear to be subtle, with some only detected in specific regions and no corresponding cognitive deficits (and few adverse symptoms) observed. However, further research is required to fully understand their clinical significance. Overall, our findings suggest that non-concussive impacts have the potential to elevate metabolism and alter neuron/glia integrity, particularly in mid- to posterior- brain regions of male soccer players. These observations substantiate suggestions that individuals should exercise caution when performing repeated non-concussive impacts in sport.

## Supplementary Information

Supplementary material 1.

## Data Availability

The data that support the findings of this study are available from the corresponding author, upon reasonable request.

## Abbreviations

^1^H-MRS: Proton magnetic resonance spectroscopy
bFFE: Balanced fast field echo
BOLD: Blood-oxygen-level-dependent
CBF: Cerebral blood flow
CNS: Central nervous system
CRT: Concussion recognition tool
CV: Coefficient of variation
dlPFC: Dorsolateral prefrontal cortex
DREAM: Dual refocusing echo acquisition mode
DWI: Diffusion weighted imaging
EEG: Electroencephalography
EPT: Electrical properties tomography
FA: Fractional anisotropy
FBA: Fixel-based analysis
FC: Fibre cross section
FD: Fibre density
FDC: Fibre density + cross section
FDR: False discovery rate
fMRI: Functional magnetic resonance imaging
FWE: Family wise-error
FWHM: Full width at half maximum
GFAP: Glial fibrillary acidic protein
Glx: Glutamate/glutamine
HR: Heart rate
ICA: Independent components analysis
IL: Interleukin
LLOQ: Lower limit of quantification
M1: Primary motor cortex
MCP-1: Monocyte chemoattractant protein-1
MD: Mean diffusivity
mI: Myo-inositol
MNI: Montreal Neurological Institute
MRI: Magnetic resonance imaging
MRS: Magnetic resonance spectroscopy
mTBI: Mild traumatic brain injury
NeuRA: Neuroscience Research Australia
Nf-L: Neurofilament light
PAL: Paired associate learning
pCASL: Pseudo continuous arterial spin labelling
PLA: Peak linear acceleration
PRA: Peak rotational acceleration
RCT: Randomised controlled trial
RF: Radiofrequency
rs-fMRI: Resting-state functional magnetic resonance imaging
SHT: Soccer heading task
sLASER: Semiadiabatic Localization by Adiabatic SElective Refocusing
SNR: Signal-to-noise ratio
SPM: Statistical parametric mapping
SWM: Spatial working memory
TARQUIN: Totally automatic robust quantitation in NMR
tCho: Total choline
tCr: Total creatine
TE: Echo time
tNAA: Total *N*-acetylaspatate
TR: Repetition time
U_SG_: Urine specific gravity
VAPOR: VAriable power and optimized relaxations
