# Supplementary material for: The Acute Effects of Non-concussive Head Impacts on Brain Microstructure, Chemistry and Function in Male Soccer Players: A Pilot Randomised Controlled Trial
Source: Sports Med Open. 2025 Jun 18;11:77. doi: 10.1186/s40798-025-00867-0 (PMC12176718; doi:10.1186/s40798-025-00867-0)
Supplement: Supplementary file 1 — Supplementary material 1. [file 40798_2025_867_MOESM1_ESM.docx]

Supplementary Files

| **Journal:** | Sports Medicine - Open |
| --- | --- |
| **Article:** | The acute effects of non-concussive head impacts in sport on brain microstructure, chemistry and function in male soccer players: A pilot randomised controlled trial. |
| **Authors:** | Nathan Delang^1,2,3^, Rebecca V. Robertson^5,6^, Fernando A. Tinoco Mendoza^5,6^, Luke A. Henderson^5,6^, Caroline D. Rae^4,7^, Stuart J. McDonald^8,9^, Ben Desbrow^1^, Christopher Irwin^1^, Aimie L. Peek^6^, Elizabeth A. Cairns^5,10,11^, Paul J. Austin^5,6^, Michael A. Green^4,12^, Nicholas W. Jenneke^6^, Jun Cao^4,13^, William T. O’Brien^8^, Shane Ball^14,17^, Michael E. Buckland^5,6,15^, Katherine Rae^16^, Iain S. McGregor^3,5,10^, & Danielle McCartney^3,5,10^. |
| **Affiliations:** | ^1^School of Health Sciences and Social Work, Griffith University, Gold Coast, Queensland, Australia  ^2^Queensland Academy of Sport, Nathan, Queensland, Australia  ^3^School of Psychology, Faculty of Science, The University of Sydney, Sydney, New South Wales, Australia  ^4^Neuroscience Research Australia, Randwick, New South Wales, Australia  ^5^Brain and Mind Centre, The University of Sydney, Sydney, Australia  ^6^School of Medical Sciences (Neuroscience), The University of Sydney, Sydney, Australia  ^7^School of Psychology, The University of New South Wales, Kensington, New South Wales, Australia  ^8^Central Clinical School, Department of Neuroscience, Monash University, Melbourne, Victoria, Australia  ^9^Alfred Hospital, Department of Neurology, Melbourne, Victoria, Australia  ^10^Lambert Initiative for Cannabinoid Therapeutics, The University of Sydney, Sydney, New South Wales, Australia  ^11^Sydney Pharmacy School, The University of Sydney, Sydney, New South Wales, Australia  ^12^School of Clinical Medicine, Faculty of Medicine and Health, The University of New South Wales, Kensington, New South Wales, Australia  ^13^School of Biomedical Sciences, The University of New South Wales, Kensington, New South Wales, Australia  ^14^School of Health Sciences (Medicine & Health), The University of Sydney, Sydney, New South Wales, Australia  ^15^Department of Neuropathology, Royal Prince Alfred Hospital, Camperdown, New South Wales, Australia  ^16^The Sports Clinic, The University of Sydney, Sydney, New South Wales, Australia  ^17^Physical Preparation Department, Sydney Uni Sport and Fitness, Sydney, NSW, Australia |
| **Corresponding Author:** | Nathan Delang. E-mail: n.delang@griffith.edu.au |

This file contains:

| **Page 2** | **Supplementary File S1.** | Eligibility Criteria. |
| --- | --- | --- |
| **Page 3** | **Supplementary File S2.** | MRSinMRS Acquisition and Analysis Checklist. |
| **Page 6** | **Supplementary File S3.** | Overlays of significant clusters derived from an independent components analysis of resting-state functional magnetic resonance imaging scans showing six resting brain networks. |
| **Page 7** | **Supplementary File S4.** | Condition characteristics. |
| **Page 8** | **Supplementary File S5.** | Example axial diffusion image overlaid with a) the fibre orientation distribution (FOD) for each fixel in the study template (with zoomed inset to show example FODs), b) a tractogram coloured by the standard red-green-blue (RGB) code indicating preferred white matter fibre bundle direction, c) the fibre density cross-section (FDC) map indicating fixel significance between heading and control group, and d) the associated effect size for the FDC measure. |
| **Page 9** | **Supplementary File S6.** | Electroencephalography data across four frequency bands and conditions. |
| **Page 10** | **Supplementary File S7.** | Cognitive function data and serum inflammatory markers across conditions and time. |
| **Page 12** | **Supplementary File S8.** | The frequency with which participants reported possible (non-specific) concussion symptoms before and after completing the SHT. |
| **Page 13** | **Supplementary File S9.** | Additional statistical analyses examining carryover effects of plasma glial fibrillary acidic protein (GFAP) concentrations and cognitive function metrics. |

Supplementary File S1: Eligibility Criteria

The following *inclusion criteria* were applied – note that asterisked (*) criteria was assessed by a medical doctor; the remaining criteria were assessed by the trial coordinator:

1. Healthy individuals aged between 18–35 years*
2. ≥5 years of soccer heading experience; that is, ≥5 full seasons of play and(or) training (with heading) the most recent of which was completed ≤2 years ago
3. Proficient in English and able to provide informed consent

Participants also needed to be confident that they could execute the SHT. This was assessed by showing individuals video footage of the soccer heading task and asking them whether they believed they could perform the task safely and successfully (‘Yes’ or ‘No’).

For the purpose of this study, “healthy” was defined as the absence of an uncontrolled, physical health condition as assessed by a medical doctor (i.e., via a verbal medical history and physical examination). Specific criteria for mental health conditions are set out below.

The following *exclusion criteria* were applied – again, note that asterisked (*) criteria were assessed by a medical doctor; the remaining criteria were assessed by the trial coordinator and(or) an investigator:

(a) A self-reported head, neck, face and/or eye injury (including concussion; as assessed via questions 2 and 3 of the Concussion History Questionnaire or at the medical doctor’s discretion) in the last 12 months*

(b) A current, self-reported injury (any type) that requires some degree of medical intervention (e.g., RICE, pain-relief medication)

(c) A self-reported history of neurological disorders (e.g., seizure disorders, closed head injuries with loss of consciousness >15-minutes, spinal cord injury, stroke)*

(d) Implantation of a cochlear device, cardiac pacemaker, intracardiac lines, medical fusion device, neurostimulator or metal plates (in the head)*

(e) Use of dental braces or eyeglasses (contact lenses permitted)

(f) A contraindication to MRI (that is not already an exclusion criterion) (e.g., body and(or) facial piercings that cannot easily be removed, a drug infusion pump that cannot easily be removed) as determined by the radiographer using the MRI Safety Checklist (Note: a ‘Yes’ response on this checklist is not itself grounds for exclusion; the radiographer will review the checklist and determine eligibility).

(g) A history of a major psychiatric disorder within the previous 12 months, as per the Diagnostic and Statistical Manual of Mental Disorders (DSM)-V criteria or at the medical doctor’s discretion, except, mild to moderate depression (score <20 on the Beck Depression Inventory [BDI]) or mild to moderate anxiety (score <16 on the Beck Anxiety Inventory [BAI])*

(h) A history of attempted suicide or current suicide ideation as determined by a score >0 on Question 9 of the Patient Health Questionnaire (PHQ)-9

(i) Inability to avoid soccer heading or any other activity involving head impacts (e.g., contact sports) 7-days prior to and while participating in this project

(j) Inability to refrain from consuming alcohol (24 hours) and caffeine (12 hours) prior to each experimental trial

(k) Inability to refrain from using anti-inflammatory medications (4-days) prior to each experimental trial (e.g., non-steroidal anti-inflammatories [NSAIDs], such as Ibuprofen, Naproxen, Diclofenac; and corticosteroids, such as Prednisolone).

(l) Inability to refrain from using central nervous system (CNS) active drugs (i.e., cocaine, cannabis, amphetamines, methamphetamine, benzodiazepines, methadone, opioids, oxycodone, barbiturates) 7-days prior to and while participating in this project as confirmed by a negative urine drug screen (UDS) (DrugCheck® NxStep Onsite Urine Test Cup)

(m) Pregnant or lactating. All female volunteers of childbearing potential will be required to complete a urine pregnancy test (Human Chorionic Gonadotrophin [hCG] Cassette, Alere^TM^). Females of childbearing potential must also agree to use a reliable form of contraception while participating in this project.

(n) Current use of platelet active agents such as aspirin, ticagrelor, clopidogrel and prasugrel, warfarin and direct oral anticoagulants (DOACs)

Supplementary File S2: MRSinMRS Acquisition and Analysis Checklist

| Site: Neuroscience Research Australia (NeuRA), Randwick, NSW, Australia | |
| --- | --- |
| 1. Hardware |  |
| a. Field strength [T] | 3T |
| b. Manufacturer | Phillips |
| c. Model (software version if available) | Ingenia CX |
| d. RF coils: nuclei (transmit/ receive), number of channels, type, body part | 32 channel ^1^H head coil |
| e. Additional hardware | N/A |
| 2. Acquisition |  |
| a. Pulse sequence | semiadiabatic Localization by Adiabatic SElective Refocusing (sLASER) |
| b. Volume of Interest (VOI) locations | Left hemisphere dorsolateral prefrontal cortex (dlPFC) and hemisphere contralateral to the athlete's dominant foot in primary motor cortex (M1) |
| c. Nominal VOI size | 15 mm^3^ |
| d. Repetition Time (TR), Echo Time (TE) | TR = 5000ms, TE = 31ms (dlPFC) and 33ms (M1) |
| e. Total number of acquisitions per spectrum | 64 averages |
| f. Additional sequence parameters | 2000Hz, 1024 data points |
| g. Water Suppression Method | VAriable Power and Optimized Relaxations delays (VAPOR) |
| h. Shimming Method, reference peak, and thresholds for “acceptance of shim” chosen | Vendor-supplied auto-shimming; second-order; FWHM <15 Hz accepted |
| i. Triggering or motion correction method | Not applicable |
| 3. Data analysis methods and outputs | |
| a. Analysis software | TARQUIN |
| b. Processing steps deviating from quoted reference or product | Pre-processing consisted of eddy current correction, lipid filtering, automatic referencing water residual removal using Hankel singular value decomposition, zero-order phase correction, and automatic referencing using zero filling. |
| c. Output measure | Water-referenced metabolite levels using Tarquin default processing. |
| d. Quantification references and assumptions, fitting model assumptions | Tarquin default basis set |
| 4. Data Quality |  |
| a. Reported variables | SNR, Metabolite Linewidth |
| b. Data exclusion criteria | Linewidth (FWHM) > 15 Hz; SNR < 5; Heat map’s visually indicating poorly placed voxels |
| c. Quality measures of postprocessing Model fitting | Metabolite Linewidth (dlPFC: 4.4±0.8, range: 3.9 – 6.4; and M1: 5.1±2.8, range: 3.9 – 14.1),  SNR (dlPFC: 17.2±4.4, range: 8.3 – 27.3; and M1: 23.0±4.1, range: 16.1 – 34.5) |
| d. Sample Spectrum | i) dlPFC 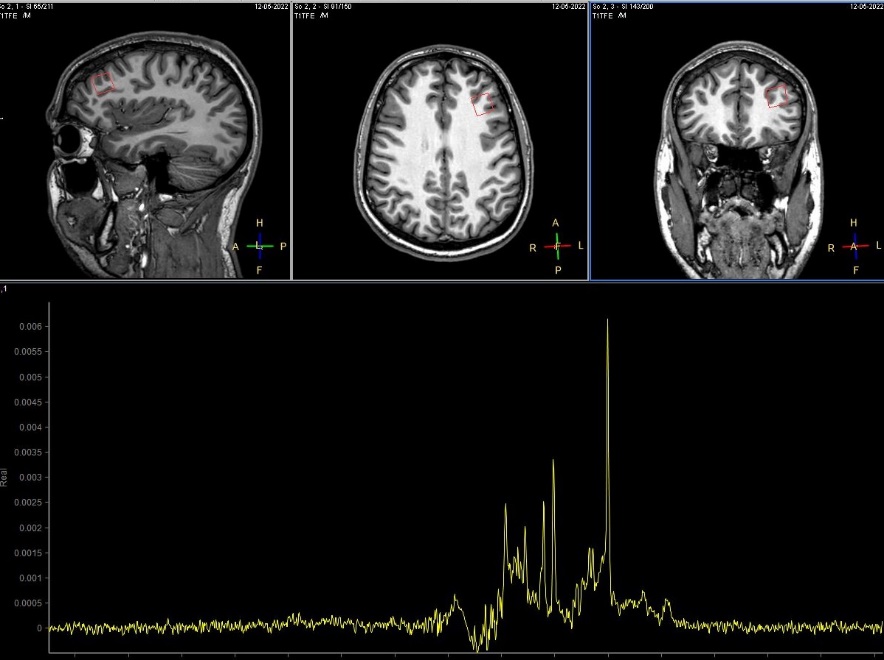  ii) M1  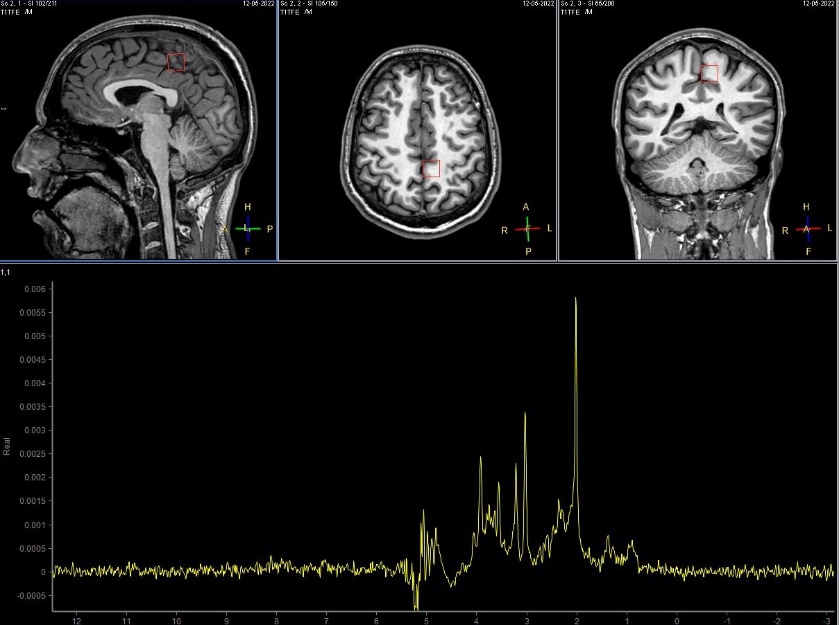 |
| e. Heat maps of voxel placement | i) dlPFC (*n*=15)  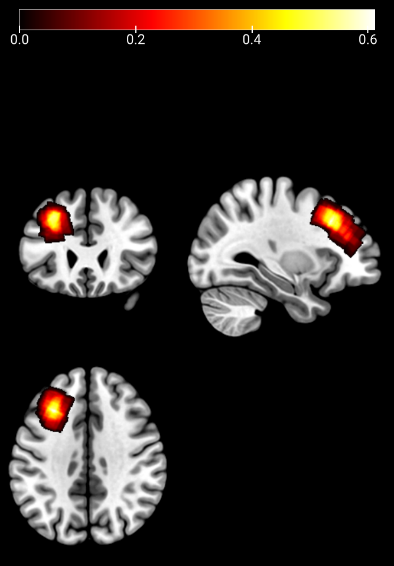  ii) M1 (*n*=15) iii) M1 (*n*=14)  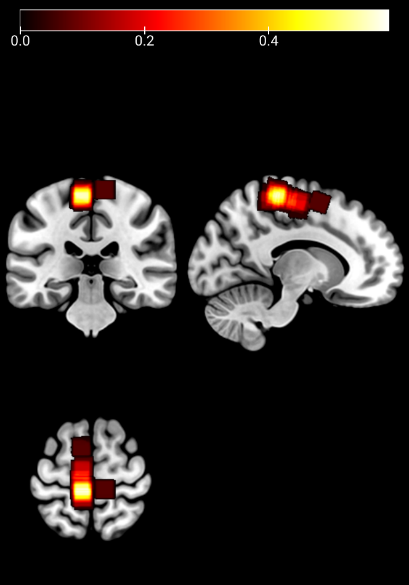 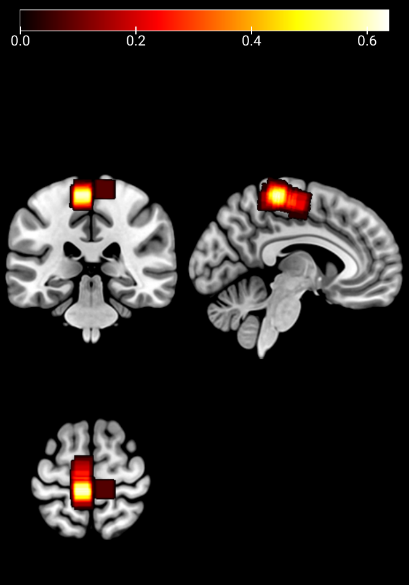 |
| Tissue Parcellation | No differences in grey or white matter fractions between Treatments were observed:  i) dlPFC (GM: Heading 42±7%, Kicking 42±7% *p*=0.66, WM: Heading 55±8%, Kicking 56±8%, *p*=0.67)  ii) M1 (GM: Heading 30±4%, Kicking 31±6%, *p*=0.56, WM: Heading 64±6%, Kicking 62±7%, *p*=0.17) |

Values are provided as mean±SD.

Supplementary File S3: Overlays of significant clusters derived from an independent components analysis of resting-state functional magnetic resonance imaging scans showing six resting brain networks.


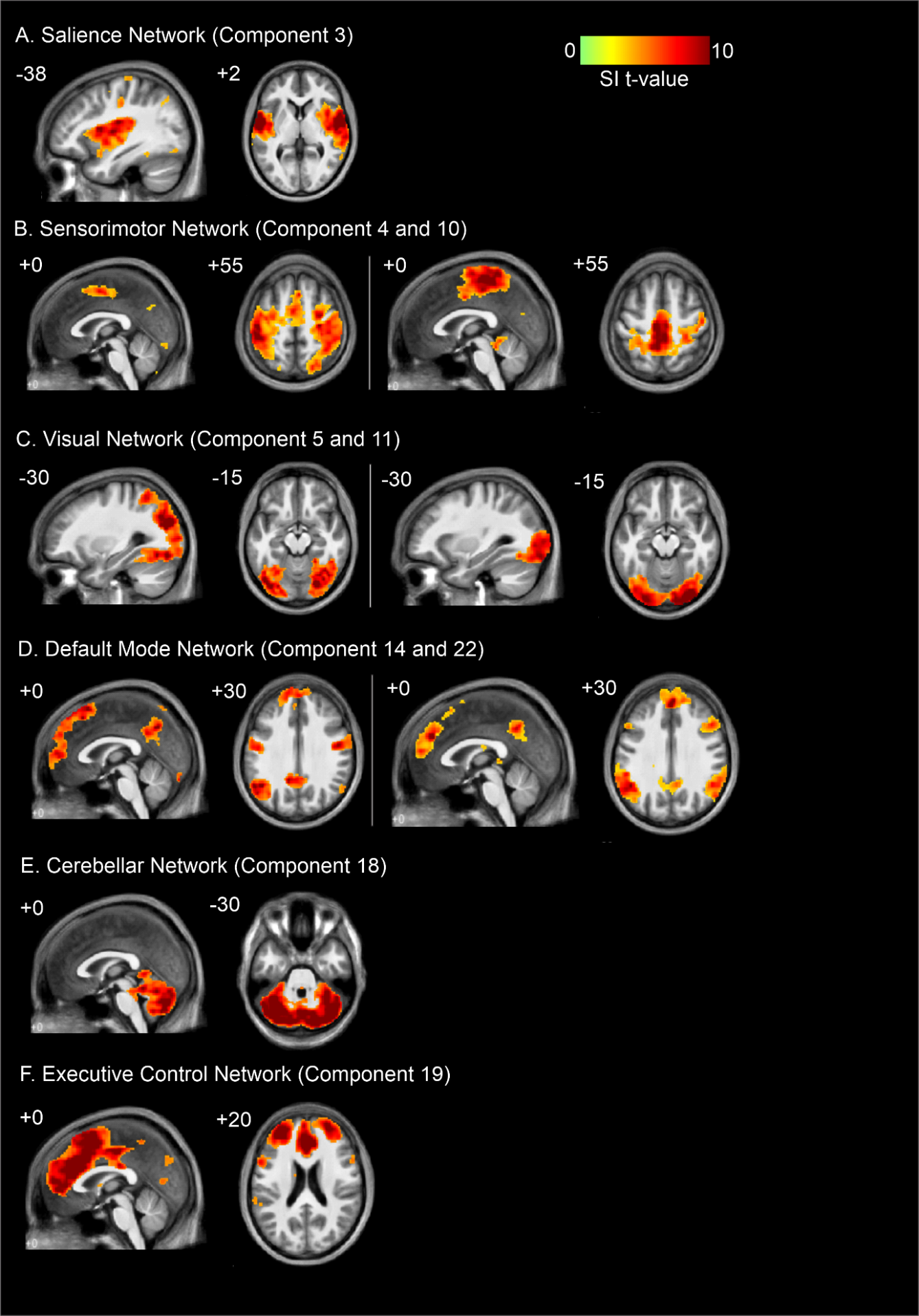


Areas are overlaid onto axial slices of a mean T1-weighted anatomical taken from all 15 participants. Location of each axial slices in Montreal Neurological Institute space are indicated at the top left of each slice. Abbreviations: SI – signal intensity

Supplementary File S4: Condition characteristics

|  | Average Linear Acceleration (g) | | Average Rotational Acceleration (rad/s^2^) | | Subjective: How strong the header felt^a^ | Subjective: How well the header was performed^a^ | Average Heart Rate   (beats/min) | |
| --- | --- | --- | --- | --- | --- | --- | --- | --- |
| ID | Heading | Kicking | Heading | Kicking | Heading | Heading | Heading | Kicking |
|  |  |  |  |  |  |  |  |  |
| 1 | 13.5±6.0 | 0±0 | 959±495 | 0±0 | 2.5 (2) | 0.5 (4) | NR | 105±7 |
| 2 | 15.2±5.1 | 0±0 | 1602±516 | 0±0 | 4 (4) | 3 (2.3) | 85±7 | 83±7 |
| 3 | NR | NR | NR | NR | 3 (2) | 1.5 (3.3) | 81±8 | 81±9 |
| 4 | 13.5±5.0 | 0±0 | 1412±636 | 0±0 | 3 (1) | 2 (5) | 60±4 | 63±3 |
| 5 | 15.6±4.3 | 0±0 | 1360±609 | 0±0 | 3 (1) | 2 (3) | 100±8 | 84±9 |
| 6 | 13.2±4.6 | 0±0 | 1277±484 | 0±0 | 3 (1) | 2 (3) | 68±7 | 69±5 |
| 7 | 16.5±5.5 | 0±0 | 1125±489 | 0±0 | 3 (1.25) | 1.5 (3.8) | 105±6 | 95±4 |
| 8 | 14.2±5.9 | NR | 957±358 | NR | 3 (2) | 0 (3) | 74±4 | 75±6 |
| 9 | 15.6±3.6 | 0±0 | 1453±499 | 0±0 | 3 (1.25) | 0 (4) | 75±5 | 65±3 |
| 10 | 17.5±6.2 | NR | 1249±783 | NR | 3 (0.25) | 2 (3.5) | 90±9 | 90±7 |
| 11 | 12.9±2.9 | 0±0 | 954±500 | 0±0 | 3 (1) | 2 (2.3) | 73±4 | 75±4 |
| 12 | 20.8±4.97 | NR | 1230±392 | NR | 3 (1) | 1.5 (2.3) | 76±7 | NA |
| 13 | 19.8±4.9 | 0±0 | 1365±733 | 0±0 | 3 (1) | 0.5 (5.5) | 80±4 | 81±5 |
| 14 | 19.7±7.6 | NR | 1538±957 | NR | 2.5 (2) | 0 (4) | 73±3 | 65±3 |
| 15 | 14.6±4.6 | 0±0 | 1364±561 | 0±0 | 2 (1.25) | -1 (3.3) | 88±8 | 68±6 |
|  |  |  |  |  |  |  |  |  |
| Mean±SD | 15.8±5.6 | 0±0 | 1271±602 | 0±0 | 3 (2) | 1 (4) | 81±12 | 79±12 |

^a^Participants were asked to rate the ‘strength’ of each header on a 5-point scale (1 = ‘very low’; to 5 = ‘very high’) and how ‘well’ they performed each header on an 11-point scale (-5 = ‘very poorly’; to +5 = ‘very well’). Abbreviations: IQR – interquartile range, NR – not recorded due to technical difficulties, SD – standard deviation. Values are provided as mean±SD or median (IQR).

Supplementary File S5. Example axial diffusion image overlaid with a) the fibre orientation distribution (FOD) for each fixel in the study template (with zoomed inset to show example FODs), b) a tractogram coloured by the standard red-green-blue (RGB) code indicating preferred white matter fibre bundle direction, c) the fibre density cross-section (FDC) map indicating fixel significance treatments, and d) the associated percentage effect size for the FDC measure relative to Kicking.


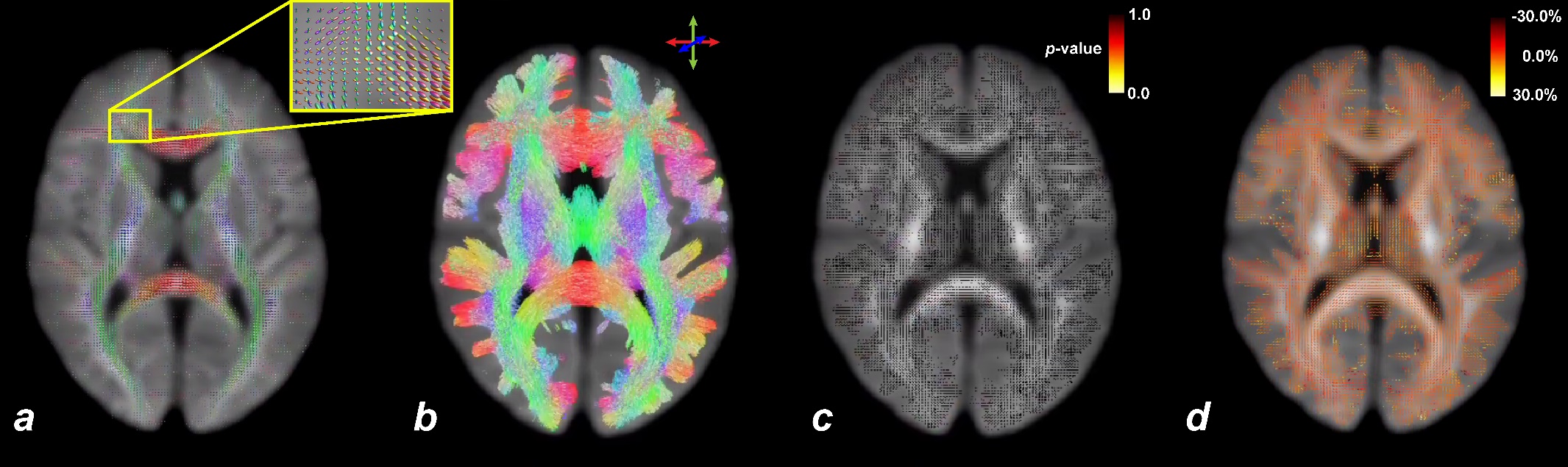


Supplementary File S6: Electroencephalography data across four frequency bands and conditions.

| **Frequency Band** | **Frequency (Hz)** | **Power (log[µV^2^])** | | ***p* value** | **Hedges’ g** |
| --- | --- | --- | --- | --- | --- |
|  |  | **Kicking** | **Heading** |  |  |
| Infraslow | 0.03-0.06 | 1.34±1.91 | 1.11±0.81 | 0.67 | -0.14 |
| Theta | 5.0-8.0 | -6.14±1.08 | -6.16±0.70 | 0.92 | -0.02 |
| Alpha | 9.0-12.0 | -5.39±0.97 | -5.63±0.74 | 0.15 | -0.23 |
| Beta | 13.0-25.0 | -7.71±0.84 | -7.78±0.50 | 0.69 | -0.09 |

Values are provided as mean±SD.

Supplementary File S7: Cognitive function data and serum inflammatory markers across conditions and time.

|  |  | Kicking | | | |  | Heading | | | |  | Interaction |
| --- | --- | --- | --- | --- | --- | --- | --- | --- | --- | --- | --- | --- |
|  |  | Pre | 0hrs Post | 2.5hrs Post | 24hrs Post |  | Pre | 0hrs Post | 2.5hrs Post | 24hrs Post |  | *p* value |
| Cognitive Function |  |  |  |  |  |  |  |  |  |  |  |  |
| PAL – Adjusted Errors (n) |  | 1.5 (5.5) | 4.0 (6.5) | 2.5 (3.0) | 2.0 (6.0) |  | 4.0 (6.0) | 2.0 (3.5) | 5.0 (4.5) | 3.0 (3.5) |  | 0.61 |
| PAL – Attempts (n) |  | 5.0 (2.0) | 6.0 (2.0) | 5.0 (1.0) | 5.0 (2.0) |  | 6.0 (1.0) | 5.0 (1.8) | 5.5 (1.8) | 5.0 (1.0) |  | 0.91 |
| SWM – Errors (n) |  | 0.0 (0.0) | 0.0 (0.8) | 0.0 (0.0) | 0.0 (0.0) |  | 0.0 (1.0) | 0.0 (0.0) | 0.0 (0.0) | 0.0 (0.0) |  | NA^a^ |
| SWM – Strategy (n) |  | 2.0 (1.0) | 2.0 (0.8) | 2.0 (0.0) | 2.0 (2.0) |  | 2.0 (2.0) | 2.0 (1.0) | 3.0 (1.8) | 2.0 (1.5) |  | 0.94 |
| Inflammatory Markers (pg/mL) | |  |  |  |  |  |  |  |  |  |  |  |
| GM-CSF |  | 12.2 (105) | NA | NA | 15.2 (161) |  | 23.1 (379) | NA | NA | 17.4 (594) |  | 0.23 |
| IFNγ |  | 4.70 (9.65) | NA | NA | 4.23 (22.7) |  | 4.98 (33.9) | NA | NA | 2.99 (41.1) |  | 0.09 |
| IL-1β |  | 8.76 (13.9) | NA | NA | 10.2 (17.9) |  | 11.8 (53.0) | NA | NA | 13.5 (45.9) |  | 0.28 |
| IL-1Ra |  | 4.58 (9.54) | NA | NA | 4.72 (11.6) |  | 5.53 (17.3) | NA | NA | 7.07 (25.0) |  | 0.37 |
| IL-2 |  | 1.75 (3.14) | NA | NA | 2.43 (4.71) |  | 2.83 (11.9) | NA | NA | 2.96 (11.3) |  | 0.03^b^ |
| IL-4 |  | 0.935 (2.92) | NA | NA | 0.925 (3.53) |  | 1.22 (8.59) | NA | NA | 1.38 (17.4) |  | 0.23 |
| IL-5 |  | 4.43 (5.32) | NA | NA | 7.31 (5.71) |  | 5.96 (5.59) | NA | NA | 5.96 (8.28) |  | 0.22 |
| IL-6 |  | 0.585 (2.83) | NA | NA | 0.410 (4.65) |  | 0.680 (4.12) | NA | NA | 0.885 (6.48) |  | 0.07 |
| IL-8 |  | 4.78 (4.53) | NA | NA | 5.04 (4.87) |  | 5.71 (4.87) | NA | NA | 5.02 (3.00) |  | 0.76 |
| IL-10 |  | 2.19 (3.32) | NA | NA | 2.32 (4.57) |  | 2.53 (5.62) | NA | NA | 3.68 (4.41) |  | 0.16 |
| IL-12p40 |  | 52.7 (221) | NA | NA | 104 (227) |  | 113 (214) | NA | NA | 142 (200) |  | 0.44 |
| IL-12p70 |  | 6.20 (26.6) | NA | NA | 7.01 (40.7) |  | 4.85 (77.7) | NA | NA | 6.50 (104) |  | 0.02^b^ |
| IL-13 |  | 125 (94.0) | NA | NA | 110 (164) |  | 120 (301) | NA | NA | 110 (388) |  | 0.12 |
| MCP-1 |  | 246 (152) | NA | NA | 220 (70.1) |  | 240 (60.6) | NA | NA | 220 (102) |  | 0.46 |
| TNFα |  | 53.4 (60.4) | NA | NA | 72.7 (144) |  | 96.0 (218) | NA | NA | 94.2 (215) |  | 0.35 |

^a^These data were not analysed as few participants made errors on the SWM task. ^b^Pairwise post hoc comparisons for IL-2 and IL-12p70 concentrations did not reach statistical significance (all p’s>0.05). Abbreviations: GM-CSF – Granulocyte macrophage colony stimulating factor, IFN – interferon, IL – interleukin, IQR – interquartile range, MCP – monocyte chemoattractant protein, PAL – paired associates learning, SWM – spatial working memory. Values provided as median (IQR).

Supplementary File S8: The frequency with which participants reported possible (non-specific) concussion symptoms before and after completing the SHT.

| Concussion Symptoms |  |  | Heading |  |  |  |
| --- | --- | --- | --- | --- | --- | --- |
|  | ‘Usual’ |  | 0hrs Post | 2.5hrs Post | 4-8hrs post | 24hrs Post |
| ‘Pressure in the head’ | 0 (0%) |  | 9 (60%) | 2 (13%) | 3 (20%) | 0 (0%) |
| Headache | 3 (20%) |  | 4 (27%) | 6 (40%) | 5 (33%) | 0 (0%) |
| Nausea | 0 (0%) |  | 1 (7%) | 0 (0%) | 0 (0%) | 0 (0%) |
| Drowsiness | 3 (20%) |  | 4 (27%) | 2 (13%) | 2 (13%) | 1 (7%) |
| Sensitivity to light and/or noise | 2 (13%) |  | 1 (7%) | 3 (20%) | 1 (7%) | 0 (0%) |
| Fatigue or low energy | 4 (27%) |  | 2 (13%) | 3 (20%) | 2 (13%) | 2 (13%) |
| ‘Don’t feel right’ | 1 (7%) |  | 3 (20%) | 1 (7%) | 1 (7%) | 0 (0%) |
| More emotional and/or irritable | 1 (7%) |  | 0 (0%) | 1 (7%) | 1 (7%) | 0 (0%) |
| Sadness | 2 (13%) |  | 0 (0%) | 0 (0%) | 0 (0%) | 0 (0%) |
| Nervous or anxious | 1 (7%) |  | 0 (0%) | 0 (0%) | 0 (0%) | 0 (0%) |
| Difficulty concentrating or remembering | 1 (7%) |  | 4 (27%) | 1 (7%) | 2 (13%) | 0 (0%) |

Symptoms were evaluated (verbal ‘Yes’ or ‘No’) using Part 3 of the Concussion Recognition Tool 5. Lighter colours represent lower frequencies; darker colours represent higher frequencies. ‘Usual’ reflects participant responses to the questionnaire at enrolment, when asked whether they have experienced any of the symptoms in the last week. Abbreviations: SHT – soccer heading task.

Supplementary File S9: Additional statistical analyses examining carryover effects of plasma glial fibrillary acidic protein (GFAP) concentrations and cognitive function metrics.

‘Pre’ plasma GFAP concentrations were analysed using linear mixed effects models that included Condition Order (categorical: Kick-Head vs. Head-Kick), Session (Categorical: Session 1, Session 2) and their interaction as fixed effects, with Participant as a random effect. The residuals were neither non-normally distributed (Shapiro–Wilk test, p>0.05) nor heteroskedastic (Levene test, p>0.05). No significant main or interaction effects were observed (*p*’s>0.100).

‘Pre’ cognitive function metrics were analysed using generalised linear mixed effects models that included Condition Order (categorical: Kick-Head vs. Head-Kick), Session (Categorical: Session 1, Session 2) and their interaction as fixed effects, with Participant as a random effect. These models were fitted to a Poisson distribution, unless over-dispersed, and/or zero-inflated. In these instances, a negative binomial, zero-inflated Poisson, or zero-inflated negative binomial distribution was substituted, respectively (with both parts of the zero-inflated models containing the same fixed effects). No significant main or interaction effects were observed (*p*’s>0.100).
